# Reviewing the ecological evidence base for management of emerging tropical zoonoses: Kyasanur Forest Disease in India as a case study

**DOI:** 10.1371/journal.pntd.0009243

**Published:** 2021-04-01

**Authors:** Sarah J. Burthe, Stefanie M. Schäfer, Festus A. Asaaga, Natrajan Balakrishnan, Mohammed Mudasssar Chanda, Narayanaswamy Darshan, Subhash L. Hoti, Shivani K. Kiran, Tanya Seshadri, Prashanth N. Srinivas, Abi T. Vanak, Bethan V. Purse

**Affiliations:** 1 UK Centre for Ecology & Hydrology, Edinburgh, United Kingdom; 2 UK Centre for Ecology & Hydrology, Wallingford, United Kingdom; 3 ICAR-National Institute of Veterinary Epidemiology and Disease Informatics, Bengaluru, India; 4 Department of Health and Family Welfare Services, Government of Karnataka, Shivamogga, India; 5 ICMR-National Institute for Traditional Medicine, Belgavi, India; 6 Vivekananda Gorukana Kalyana Kendra (VGKK), Chamarajanagar, India; 7 Ashoka Trust for Ecology and the Environment, Bengaluru, India; 8 DBT/Wellcome Trust India Alliance Fellow, Hyderabad, India; 9 Institute of Public Health, Bangalore, India; 10 School of Life Sciences, University of KwaZulu-Natal, Durban, South Africa; Beijing Children’s Hospital, Capital Medical University, CHINA

## Abstract

Zoonoses disproportionately affect tropical communities and are associated with human modification and use of ecosystems. Effective management is hampered by poor ecological understanding of disease transmission and often focuses on human vaccination or treatment. Better ecological understanding of multi-vector and multi-host transmission, social and environmental factors altering human exposure, might enable a broader suite of management options. Options may include “ecological interventions” that target vectors or hosts and require good knowledge of underlying transmission processes, which may be more effective, economical, and long lasting than conventional approaches. New frameworks identify the hierarchical series of barriers that a pathogen needs to overcome before human spillover occurs and demonstrate how ecological interventions may strengthen these barriers and complement human-focused disease control. We extend these frameworks for vector-borne zoonoses, focusing on Kyasanur Forest Disease Virus (KFDV), a tick-borne, neglected zoonosis affecting poor forest communities in India, involving complex communities of tick and host species. We identify the hierarchical barriers to pathogen transmission targeted by existing management. We show that existing interventions mainly focus on human barriers (via personal protection and vaccination) or at barriers relating to Kyasanur Forest Disease (KFD) vectors (tick control on cattle and at the sites of host (monkey) deaths). We review the validity of existing management guidance for KFD through literature review and interviews with disease managers. Efficacy of interventions was difficult to quantify due to poor empirical understanding of KFDV–vector–host ecology, particularly the role of cattle and monkeys in the disease transmission cycle. Cattle are hypothesised to amplify tick populations. Monkeys may act as sentinels of human infection or are hypothesised to act as amplifying hosts for KFDV, but the spatial scale of risk arising from ticks infected via monkeys versus small mammal reservoirs is unclear. We identified 19 urgent research priorities for refinement of current management strategies or development of ecological interventions targeting vectors and host barriers to prevent disease spillover in the future.

## Author summary

Zoonotic diseases (caused by pathogens passed between animals and people) disproportionately affect tropical communities. Management to prevent human cases is often hindered by a poor ecological understanding of zoonotic disease systems, which may involve complex communities of vector (e.g., ticks) and host species involved in pathogen transmission. Thus, management usually focuses on human medical treatments such as vaccination. However, there may be other alternative interventions targeted at vectors or wildlife hosts which may be more effective, economical, or long lasting. Furthermore, more conventional human-focused intervention strategies could be improved through better ecological understanding of the disease system. Here, we refine a new framework developed to identify barriers in the disease system that a pathogen needs to overcome in order to spillover to cause human disease cases, to include additional barriers pertinent to vector-borne pathogens. We apply this refined framework to Kyasanur Forest Disease (KFD), a potentially deadly tick-borne infection from India which affects poor forest communities in the Western Ghats and demonstrate how it can be used to identify key knowledge gaps that that need to be addressed in order to evaluate the effectiveness of existing management and to design new interventions.

## Introduction

Zoonotic diseases disproportionately affect tropical communities, resulting in 26% of disability-adjusted life years lost to infectious diseases in lower middle-income countries [1–3]. Their burdens and impacts are increasing worldwide due to wide-ranging sociopolitical, ecological, and environmental changes [4]. Most zoonotic diseases have complex transmission cycles involving communities of vector and animal hosts, with disease dynamics highly dependent on host ecology and evolutionary biology [5,6]. Further complexity arises since human behaviour and ecosystem use alters exposure to infected vectors and hosts [7], making it challenging to predict human infection risk and develop effective control strategies. This complexity is highlighted in the global “One Health” initiative, which recognises the interconnectedness of human and animal health and the environment and recommends a coordinated, interdisciplinary, cross-sectoral approach to management of zoonotic diseases [8,9]. Despite this, many zoonotic disease control programmes utilise interventions that are focused on humans (e.g., vaccination and preventative drug treatment). Such conventional interventions are applied without understanding or consideration of the underlying ecological complexity and environmental settings in which spillover to humans occurs (spillover defined as transmission of a pathogen from a vertebrate animal to a human [5,10]). This is particularly true of neglected zoonoses, for example, rabies, echinococcosis, leishmaniasis, and leprosy that primarily affect poor and marginalised populations in low-resource settings. Neglected zoonoses are defined as those that receive less attention and funding (for both research and interventions) compared to diseases such as malaria, tuberculosis, and HIV/AIDS, leading not only to vast underreporting but also to poor understanding of the disease systems [5,11,12]. Integrating ecological and evolutionary understanding of multi-vector and multi-host transmission, human and environmental factors into disease control policy is considered essential for reducing the impact and probability of emergence of zoonotic diseases [5,6,10]. However, there are widespread examples of effective disease control being hampered by a poor ecological evidence base or limited application of existing evidence into policy and practice (see reviews in [5,13]).

**Fig 1:**
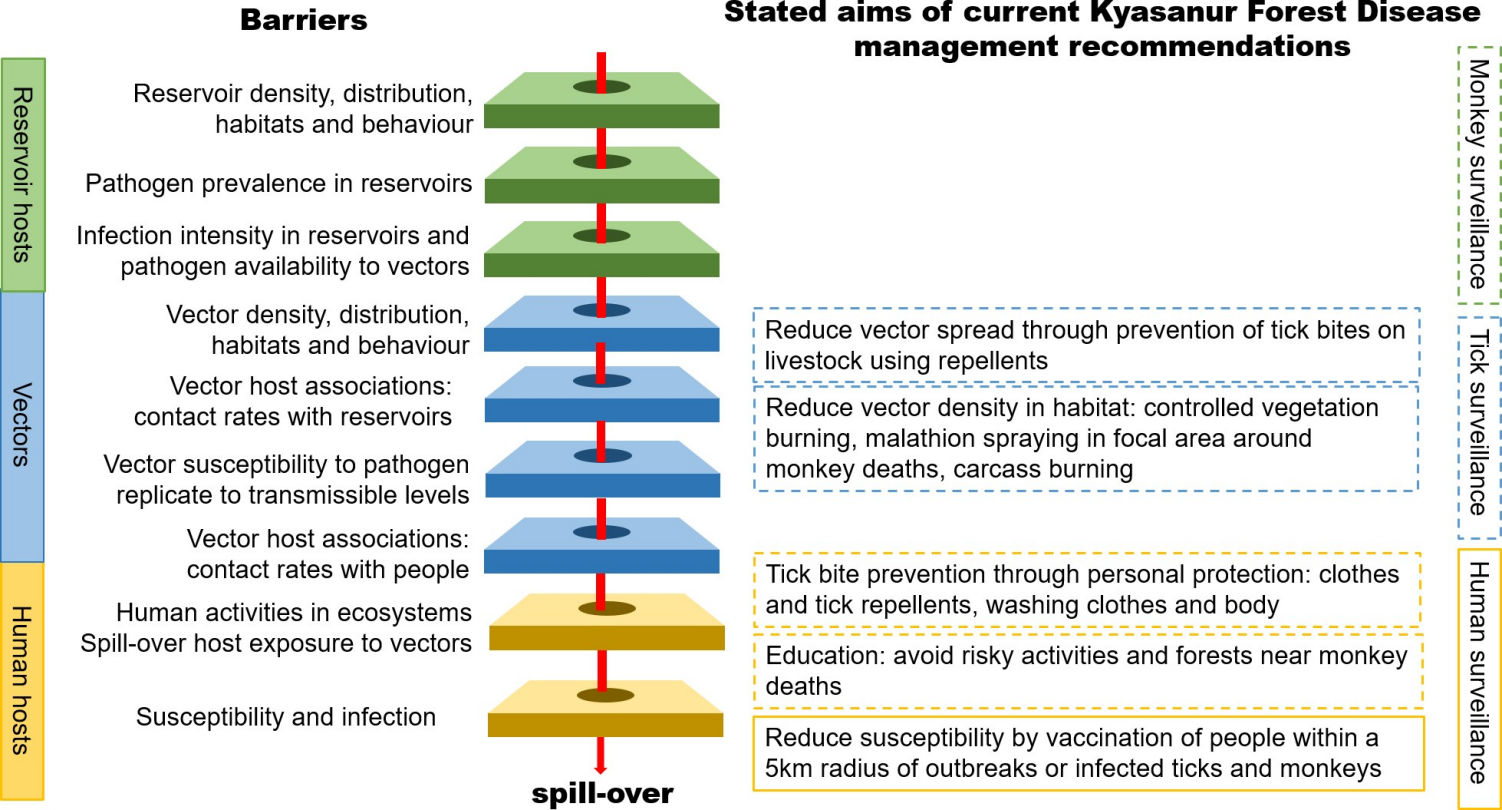
A schematic of the hierarchical barriers to spillover of vector-borne zoonotic diseases to humans, extending the framework set out in [10,14]. Management interventions may reduce or prevent spillover by targeting these barriers, with green layers representing reservoir hosts, blue representing the environment and vectors, and yellow the spillover hosts. Current KFD management shown on the right-hand side mainly targets the final 2 barriers associated with the spillover hosts, aiming to reduce human exposure and susceptibility to infection. The dotted outlines of boxes indicate where the empirical evidence for impacts of management interventions is particularly incomplete. Surveillance activity, currently conducted for KFDV in people, ticks, and monkeys informs these interventions, with dotted outlines indicating where strategies could be refined to better target interventions. KFD, Kyasanur Forest Disease; KFDV, Kyasanur Forest Disease Virus.

New frameworks have recently been developed that identify the hierarchical series of barriers that a pathogen needs to overcome for spillover from a wildlife reservoir into a human host to occur. Such barriers could then be targeted by management interventions to prevent human disease ([10,14], Fig 1). These frameworks demonstrate how a broad range of interventions could be developed to complement conventional approaches which target humans such as vaccination and drug treatment [10]. In contrast to conventional interventions, ecological interventions are defined as those that consider the ecological context in which human spillover occurs and which harness improved understanding of the disease system ecology in order to manage the underlying transmission process [10]. An example would be restoration of natural enemy populations through habitat creation or management in order to reduce maintenance host populations. This strengthens the important barrier against having sufficient densities of reservoir hosts necessary for effective pathogen transmission [10]. Such ecological interventions may lead to more targeted, long-term, effective, and economical approaches to managing human disease cases because they aim to disrupt underlying transmission processes. A good example of the effectiveness of ecological versus conventional interventions is in the case of Nipah virus control in Asia. Here, knowledge of transmission mechanisms, reservoir and amplifying hosts, and human social factors driving infection risk led to the development of ecological intervention strategies and a move away from conventional approaches such as culling of wildlife and domestic animal hosts. *Pteropus* spp. of bats are known to be maintenance hosts for the Nipah virus, but pigs are amplifying hosts [15–17]. Human spillover arises through direct ingestion of fruit contaminated by Nipah virus–shedding bats or by pigs, which are also infected via ingestion of infected fruit or contamination with bat faeces or urine [18–20]. Based on this knowledge, ecological interventions that target the human spillover barrier of human host exposure are being practiced and developed including surveillance in pigs in areas where pig farming overlaps with *Pteropus* spp. distributions to facilitate early intervention, restrictions on fruit trees near pig farms, the use of bamboo skirts over sap collection pots to prevent contamination, and education and measures to prevent human consumption of contaminated plant products [21–23].

Current frameworks for identifying hierarchical barriers to spillover [10] are particularly suited to directly transmitted pathogens. However, a large proportion of zoonotic diseases are vector-borne, and refinement is needed to identify additional barriers applicable to such diseases. To inform the development and operationalisation of such frameworks in real-world settings, we focus on a case study: an emerging, tick-borne zoonotic pathogen in India.

India has been identified as a global hotspot of zoonotic emerging disease risk, alongside other tropical forest regions in Central Africa, South America, and Asia, because of the high levels of deforestation and land-use change, high biodiversity and spatial overlap between wildlife and human populations, high human and livestock population densities, and also the low performance of health systems [24,25]. Moreover, India ranks high both in terms of the diversity of endemic and emerging zoonotic diseases, for example, rabies, anthrax, leishmaniasis, and leptospirosis, and the resulting economic and health burden [26–29]. Kyasanur Forest Disease Virus (KFDV) is a tick-borne virus (family Flaviviridae, genus *Flavivirus*) causing debilitating and potentially fatal haemorrhagic disease (approximately 500 cases per annum, up to 10% mortality [30]) in people in the Western Ghats region of South India. Historically, Kyasanur Forest Disease (KFD) cases were restricted to a small number of districts in Karnataka state since the disease was first described in 1957 [30,31]. Human cases of KFD have increased since 2005, with a recent dramatic spread to neighbouring states of Goa, Tamil Nadu, Maharashtra, and Kerala [32]. The disease primarily affects low-income rural forest communities such as small-holder farmers, plantation and forestry workers, and tribal groups reliant on harvesting of non-timber forest products [33–35]. Household surveys of small-holder farmers and tribal groups within KFD-affected areas found that 69% of respondents (*n* = 227) were perturbed by the impact of KFD on their livelihoods, highlighting KFD as a major health issue in the region [36].

As well as affecting diverse forest users, KFDV has a broad vector and host range. The transmission cycle is complex, with various tick species involved (principally *Haemaphysalis spinigera* but infection has also been reported from an additional 8 *Haemaphysalis* species and some *Ixodes*) and multiple hosts implicated, including wild rodents and shrews, bats, monkeys, and some birds [30]. Despite some experimental evidence from laboratory studies [37,38] and modelling [39], transovarial transmission, whereby adult female ticks pass KFDV to their offspring, has never been recorded in the wild. Humans contract KFDV when bitten by an infected tick, which became infected either by feeding on a reservoir host with systemic infection or by feeding in close proximity to an infected tick before moulting and biting a human host. Humans are incidental hosts for the disease and are not involved in onward transmission [40]. Thus, like the Lyme disease agent *Borrelia burgdorferi*, KFDV is a spillover pathogen for which almost every human case represents a spillover event from a wildlife reservoir via the infected tick vector. However, we lack empirical knowledge of the role of different species of vector and hosts in the KFDV transmission cycle and how human behaviour and environmental changes like deforestation are leading to disease spillover. Monkeys, principally the black-footed grey langur (*Semnopithecus hypoleucos*) and the bonnet macaque (*Macaca radiata*), are hypothesised to act as amplifying hosts, because infection has been shown to lead to high titres of circulating virus, meaning they are likely to pass infection to their ticks [41]. Cattle are not considered to amplify KFDV since they do not develop viraemia of long duration [42,43], but are hypothesised to increase tick population density through their importance as a blood meal host [44,45]. The lack of robust testing of these 2 hypotheses in the field is indicative of the significant gaps in our empirical knowledge of the ecology of the KFD system. Even though spillover of KFDV to humans has been widely linked to deforestation [31,46–48], we lack mechanistic understanding of these patterns. Empirical data collection on host–vector–pathogen interactions is restricted to disease emergence events that occurred last century in the 1960s and 1980s and has not been updated for current, further degraded forest conditions or for areas where KFD has more recently emerged.

The purpose of this review is to (1) identify current management recommendations to prevent human KFD cases and map them onto a framework of the hierarchical barriers to spillover for a vector-borne pathogen; (2) review the empirical evidence underpinning each KFD management recommendation; (3) review evidence for the effectiveness of the current management practice; and (4) identify knowledge gaps in our understanding of KFD–vector–host transmission dynamics that currently prevent evaluation of management options and development of a broader range of interventions, including ecological interventions, to prevent human cases of KFD.

## Methods

### Ethics statement

The protocols for this study were approved by the Institutional Ethics Committee of the Institute of Public Health (IPH IEC), Bangalore (Study ID, IEC-FR/04/2017) and received a Favourable Ethical Opinion from the Liverpool School of Tropical Medicine Research Ethics Committee (research protocol 17/062). All workshop and interview participants were adults and provided informed consent via email through acceptance of the workshop invitation or verbal consent for interviewees.

### Reviewing current management recommendations and empirical support: A coproduction approach

This review is part of our interdisciplinary One Health Indo–UK partnership, the MonkeyFeverRisk project (https://www.monkeyfeverrisk.ceh.ac.uk/), which together with disease managers and policy makers across the public health, animal health, and environmental sectors, aims to improve our understanding of risk factors and coproduce guidance and decision supports tools for KFD [23]. Embedded in this One Health network, we reviewed the current management practices undertaken to prevent human cases of KFD, identifying guidance documents and sources in the grey literature available to disease managers. In order to review the empirical evidence for these management practices for the KFD system, we conducted a literature search for the search term “KYASANUR” using Web of Science and PubMed for peer-reviewed literature and discussed with key stakeholders to ensure that further grey literature was not missed. In order to identify literature pertaining to the ecological evidence underpinning management practices for tick control for the main tick species involved in KFD transmission, we also conducted searches for “HAEMAPHYSALIS AND INDIA.” Where there was no direct empirical evidence for a specific management practice impacting on KFD or *Haemaphysalis* tick vectors, we searched for additional evidence from other tick-borne infections, for example, from well-studied systems such as Lyme disease. However, a full review of topics, for example, repellent and acaricide efficacy, was beyond the scope of this paper. Therefore, searches via PubMed and Web of Science were made primarily for review papers on specific topics. Search terms used to identify literature are provided in Table 1. Some recommended management practices, such as wearing protective clothing and washing of clothes to remove ticks, are well-accepted interventions that are endorsed by global health bodies such as the World Health Organization (WHO), and we cite such web pages where appropriate (Table A in S1 Appendix).

**Table 1:** Search terms used to identify literature in Web of Science and PubMed providing empirical evidence for disease ecology and transmission of KFD and for the effectiveness of current management recommendations.

Search term(s)	Number of citations identified
KYASANUR	257
HAEMAPHYSALIS AND INDIA	108
REPELLENT AND TICK AND REVIEW AND EFFICACY	32
ACARICIDE AND TICK AND REVIEW AND EFFICACY	46
VEGETATION AND TICK AND BURNING	19
CLOTHES AND WASHING AND TICK	21
CLOTHES AND PROTECTIVE AND TICK	166

KFD, Kyasanur Forest Disease.

We supplemented the review of documents and published literature on KFD management with a series of key informant interviews to ascertain views and experiences with KFD surveillance and control. Interviews were conducted (between July and August 2019) with district and taluk public health managers (*N* = 11) from Shivamogga headquarters, Sagar and Thirthahalli taluks in Karnataka, directly responsible for KFD management. The selection of interviewees was based on purposive and snowball sampling, which are advantageous techniques in situations where existing networks of relevant people are lacking. Purposeful sampling identifies a key set of people with the relevant expertise and/or experience to provide information and insights on the topic. Snowball sampling then broadens the net of potential interviewees when further relevant people are identified by the interviewees from the purposive sampling. Interviews were conducted in Kannada or English based on participant preference and were transcribed and analysed using an open thematic coding approach [49]. In addition, we also collated and analysed messages shared on our MonkeyFeverRisk WhatsApp platform, a group numbering 121 participants set up in 2018 at the request of our stakeholders to facilitate knowledge exchange on KFD between researchers and practitioners working in different sectors and affected areas. The messages reported here were specific questions posed to researchers by participants about KFD management that illustrated evidence and knowledge gaps about how environmental or wildlife management aligns with KFD management guidance (Table C in S1 Appendix). For the interview data, identified patterns and contradictions from the data were coded and organised into emergent themes. These themes were reviewed to ensure they accurately reflected the meanings evident in the data. The initial findings were noted and subsequently compared with the document review (of current management practices) based on which recommendations for effective and integrated management are made [50]. Participation was voluntary, and interviewees gave their full prior informed verbal consent before the conduct of the interviews, which lasted between 30 and 45 minutes. The interview data were anonymised to protect the confidentiality of participants. Table A in S1 Appendix outlines the main themes and exemplar quotes identified through the key informant interviews, and Table B in S1 Appendix identifies key informants.

We extended the framework of Sokolow and colleagues [10] to consider additional barriers that operate for vector-borne zoonotic diseases compared to those that are directly transmitted. Vector-borne pathogens not only have to evade the immune systems of reservoir hosts and humans to effect spillover, but also have to overcome several tissue barriers and immune responses within the body of the arthropod vector in order to survive and replicate to transmissible levels (see review by [51]). A key prerequisite for spillover of vector-borne zoonotic pathogens is that their arthropod vectors are sufficiently abundant, widespread [52], and overlap in the same habitat with key reservoirs and with people [53]. Furthermore, spillover host exposure depends on how human activities within the ecosystem interface with vector and reservoir host habitats and behaviour [54] and whether vectors prefer to bite both people and reservoirs [55]. We then mapped the existing key management recommendations identified from our review onto our new extended framework for vector-borne zoonoses.

We summarised our overall assessment of the validity of current management practices by scoring each recommendation using separate traffic light scales for the degree of empirical support and management effectiveness. For assessment of empirical support, red indicates no or poor support; amber indicates some support from observations and laboratory studies but lacking rigorous empirical data in a field setting; and green indicates good empirical support including rigorous empirical field data. For management effectiveness, red indicates that the management practice is unlikely to significantly reduce human cases of KFD; amber indicates that it is unknown whether the management practice will reduce human cases; and green indicates that the management practice will reduce human cases of KFD. Our proposed research and surveillance priorities were targeted at addressing knowledge gaps (a) to improve the existing management practices (short-term priorities); or (b) to allow more integrated or ecological interventions to be implemented in the future (long-term fundamental research priorities).

## Results

The review of existing management practices and supporting empirical evidence for KFD identified 257 sources based on the keywords “KYASANUR” and an additional 67 sources (108 citations identified in total) based on the keywords “HAEMAPHYSALIS AND INDIA” (Table 1). The numbers of citations pertaining to reviews of topics where empirical information was not specifically available for the KFD system are provided in Table 1. Two key guidance documents detailing existing management recommendations for KFD were identified by stakeholders in the grey literature, originating from the public health sector, namely the Indian National Centre for Disease Control and the Department of Health and Family Welfare Services, Government of Karnataka (DHFWS), a guidance bulletin [56] and a manual on KFD [57]. Full details of the review of empirical support for current management practices recommended for preventing human cases of KFD in the Western Ghats area of India are given in Table A in S1 Appendix. Although Table A is not included in the main text of the paper due to space constraints, we hope this detailed table will provide a key reference document for practitioners involved in KFD management. Table 2 summarises current management practices, indicating that many of the current management interventions are not well supported by empirical evidence. Of the 15 management recommendations identified, only 5 had sufficient empirical support pertinent to KFD, with a further 2 supported by empirical evidence from other disease systems (green on the traffic light assessments). Moreover, evidence was not deemed robust enough to designate any of the management practices as being highly effective, with all 15 measures being assessed as red or amber on the traffic light scale. Below, in the discussion, we summarise the validity of current KFD intervention measures and identify knowledge gaps and research priorities. We also map these research priorities onto the hierarchical barriers framework (see Table 3).

**Table 2:** Overall assessment of the validity of current management practices for KFD.

Current management recommendations for KFDV	Local empirical evidence	Evidence from other systems	Rationale for evidence score	Effectiveness of management practice	Rationale for effectiveness assessment	Recommendation
PPMs should be taken (long clothes covering neck, chest, back, and legs) before going to the forest	Green	Green	Good evidence from multiple systems that PPMs can reduce tick bites	Amber	Only effective in conjunction with application of effective repellents, washing the clothes and body, and effective checking and removal of attached ticks	PPMs should be recommended for any activity where persons may brush against vegetation that may harbour ticks, not just forests, and should include covering the feet and tucking in clothes
People living in the forest or visiting forest areas should use tick repellents (DMP oil, DEET, or local herbs) before going to the forest. Permethrin-based repellents should be used on clothing	Amber	Green	Good evidence that repellents prevent tick bites, but efficacy of locally available repellents may be poor or untested	Amber	Locally available repellents may have poor efficacy. Only effective in conjunction with appropriate clothing, washing the clothes and body, and effective checking and removal of attached ticks	Recommend applying repellents during any activity where persons may brush through vegetation that may harbour ticks, not just forests, and guidance on reapplying repellents regularly
People should wash their clothes and body with hot water and soap after returning from the forest	Amber	Green	Good evidence from other systems that washing can remove unattached ticks, but more limited local evidence and people use cold water	Amber	Only effective in conjunction with wearing of appropriate clothing, application of effective repellents, and effective checking and removal of attached ticks	Recommend that additional education is needed to inform people that washing alone will not remove attached ticks from the body
The spraying of insecticide (malathion) may be carried out in areas where monkey deaths have been reported within a radius of 50 feet around the location of the monkey death. It is also effective on forest tracks frequently visited by people for various activities	Red	Amber	May be effective over the area of spraying in the short term but effectiveness untested locally and little known about resistance	Red	Infected ticks likely to be found across broader habitats associated with monkey deaths so spraying a small area is likely ineffective. Malathion resistance may be problematic	Not recommended without empirical evidence of effectiveness and better knowledge of the scale of infection risk
Application/injection of insecticide on/into cattle can prevent ticks and the transportation of ticks from forests to dwelling premises	Amber	Amber	Acaricides can be effective at lessening tick burden on livestock (although caution needed due to resistance), but no evidence that they prevent tick movements	Red	May well prevent tick movements but no empirical evidence that cattle are associated with higher prevalence of human KFDV cases. Untested whether cattle might operate as diluting hosts for KFDV	Need more evidence before recommending as KFDV preventative measure but need to consider prevention of other tick transmitted infections too
Controlled burning of the dry leaves and bushes in the forest boundaries, premises of human habitats	Red	Amber	Conflicting evidence about the temporal scale over which this lowers tick abundance, lack of data on whether forests are main KFDV-risky habitat	Red	Unclear whether this may increase tick abundance in the longer term	Not recommended without empirical evidence of effectiveness and better knowledge of the scale of KFDV infection risk
Burning of monkey carcass	Red	Red	No empirical support that dying or dead monkeys create hotspots of infected ticks	Red	Infected ticks likely to be found across broader habitats and burning monkey carcass unlikely to be important for preventing KFD	Recommended as is a good way of disposal of carcasses which may pose a general risk to human health through disease transmission from bodily fluids. Robust postmortems and sample collection protocols needed prior to burning
Vaccination of people within a 5-km radius of cases	Green	Green	Substantial evidence that vaccination reduces human cases of KFDV	Amber	Vaccine efficacy and formulation needs to be improved. Vaccine uptake is poor as administration is painful, requires 3 initial doses, and annual boosters to confer immunity. Modelling is needed to optimise the spatial scale over which vaccination is targeted	Urgent need for a more effective vaccine with fewer doses required and better education to increase uptake. Need better understanding of the scale at which risk operates
Educate the villagers to avoid the forests areas where monkeys have died. Don’t visit the area where recent monkey death has been reported, especially an area where case of KFDV has been reported in the past	Amber		Evidence that monkeys may act as sentinels of human disease but poor empirical evidence over the mechanism and spatial scaling	Amber	If monkeys are effective sentinels then avoiding forests may help prevent human cases	Need better empirical evidence of tick habitat associations and better knowledge of the scale of infection risk. Education needed on effective PPM and risk associated with brushing against vegetation, not just in forests
Don’t bring the leaves of trees from KFDV-infected area to the village for cattle bedding material	Amber		Ticks have been found in leaf litter but survival times in such litter are unknown	Amber	May prevent the spread of infected ticks but need for better empirical evidence. Alternative sources of bedding may not be available	Need better empirical testing of the risks posed by leaf collection from different habitats, and the levels of tick infestation in leaf litter used for animal fodder and bedding. Also need more education on appropriate PPM
Don’t handle the infected monkey carcass by bare hand without personal protective equipment	Amber	Amber	Good evidence from multiple systems that protective clothing can reduce tick bites but needs to be more than wearing gloves	Amber	Only effective in conjunction with application of effective repellents, washing the clothes and body, and effective checking and removal of attached ticks. Needs to be undertaken not just when handling monkey carcasses	Monkeys should not be handled by members of the public. PPM should be recommended for any activity where persons may brush against vegetation that may harbour ticks, not just when handling monkeys
Highlighting risky activities: for example, to not sit on the ground or in bushy areas of the forest	Amber	Amber	Evidence that ticks move onto humans when they brush against vegetation, some species actively quest. Limited empirical quantification of questing behaviour in vectors associated with KFDV in the wild and of the risk associated with different habitats and human activities	Amber	Difficult to judge effectiveness without further empirical data on how activities in different habitats increase KFD risk and on tick habitat associations. Emphasis should not just be on forests without better empirical data on risk	Reasonable to keep recommendation but to expand to be aware that risk of ticks may occur in habitats other than forests and that effective PPM, use of repellents, and checking for ticks are essential
Human disease surveillance: surveillance of fever cases between December and May with sera screened for KFDV antibodies in order to target vaccination	Green	Green	Surveillance is a useful way of monitoring past and present spillover	Amber	Surveillance needs to be undertaken strategically across areas both within and out with the historical KFD regions	Recommended but improvements could be made to how surveillance effort is targeted
Tick surveillance: surveillance is undertaken within 5 km of areas where human cases were recorded in the previous year (for up to 5 years) or within 5 km of areas with current monkey deaths. Surveillance is not undertaken if current human cases are recorded	Green	Green	Surveillance of ticks can be an effective way of monitoring past and present spillover	Amber	Effectiveness difficult to judge without better empirical knowledge of KFDV infection–tick–host–habitat associations so that surveillance can be effectively targeted. Surveillance needs to be undertaken strategically across areas both within and out with the historical KFD regions with more systematic sampling of habitats and across seasons	Valuable management tool. Needs better underpinning by empirical evidence to enable better targeting of habitats and seasonality. Need additional information on hosts to be able to determine best surveillance strategies in terms of habitats and spatial scale and hosts (e.g., rodents) to target
Monkey disease surveillance: testing of dead and dying monkeys for KFDV infection	Green	Green	Monkeys are known to be amplifying hosts for the virus so are useful sentinels that may give warning of impending human infections	Amber	Stratified proactive sampling of monkeys is not undertaken, just reactive sampling of dead or dying monkeys	More stratified sampling of monkey blood for both antibodies and active infection with KFDV at sentinel sites. Better education about reporting dying/dead monkeys and faster response and sampling of monkeys and sampling for ticks around carcasses and the broader environment are recommended

Empirical support underpinning each management recommendation are assessed based on a traffic light scale at both the local level (Western Ghats of India for KFD) and also at a more global scale if evidence for this management being effective has been observed in other tick-borne disease systems (left blank if not applicable). **Red** indicates no or poor support; **amber** indicates some support from observations and laboratory studies but lacking rigorous empirical data in a field setting; and **green** indicates good support including rigorous empirical field data. Management effectiveness was also scored on a traffic light scale: **Red** indicates that the management practice is unlikely to significantly reduce human cases of KFD; **amber** indicates that it is unknown whether the management practice will reduce human cases; and **green** indicates that the management practice will reduce human cases of KFD.

DMP, dimethyl phthalate; KFD, Kyasanur Forest Disease; KFDV, Kyasanur Forest Disease Virus; PPM, personal protection measure.

**Table 3:** Key research priorities under each of the barriers that could be targeted to prevent KFD spillover to humans (Fig 1) and how these would inform and improve existing management strategies (a) and facilitate the development and future implementation of integrated, ecological interventions in the long term (b).

Research priority	(a) Refines current management or surveillance (short term)	(b) Facilitates future ecological interventions (long term)
Barrier: Preventing tick bites on people through personal protective measures		
1. Systematically review and test the efficacy of natural repellents being used against ticks by people in the Western Ghats alongside chemical repellents recommended by the Indian Government and by WHO		X
2. Develop standard assays, including in vivo and in vitro toxicity tests, to assess safety and efficacy of repellents in the laboratory and under field conditions against local tick vector species	X	X
3. Determine whether ticks survive washing and drying of clothes and then pose a risk to humans through rigorous experiments with different washing and drying regimes	X	
Barrier: Vector density, distribution, habitats, and behaviour		
4. Quantify abundance and infection rates of tick vector species across different habitats within the agroforest mosaic (integrate into stratified tick surveillance)	X	X
5. Determine whether cattle are amplifying and spreading tick species or acting to dilute infection by comparing tick burdens and KFDV infection rates on cattle, wildlife hosts, and people, in settings varying in host densities	X	X
6. Quantify abundance and infection rates of ticks found in different types of dry leaf litter, used for animal fodder and bedding, under different treatments in villages	X	X
Barrier: Vector host associations: Contact rates with people		
7. Quantify effectiveness of different acaricide formulations, doses, and frequencies of application in reducing tick burdens on cattle, for those species involved in KFDV transmission and for natural as well as chemical repellents	X	
8. Determine whether acaricide resistance is widespread in tick populations in India, in tick species involved in KFDV transmission, for acaricides applied both to animals and to the habitat		X
Barrier: Human activities in ecosystems		
9. Quantify rate of contact between people and ticks during different activities in and around the forest	X	X
Barrier: Pathogen prevalence, infection intensity in reservoirs, and pathogen availability to vectors		
10. Determine role of dead and dying monkeys in generating hotspots of transmission: quantify burdens, age structure, feeding history, and infection rates of ticks found on dead and dying monkeys, small mammals, and nearby habitats and people at the same time as measuring host infection levels	X	X
11. Determine role of live monkeys in transmission through infection of larvae via systemic circulation and/or supporting co-feeding between nymphs and larvae: quantify burdens, age structure, feeding history (via blood meal analysis), and infection rates of ticks found on live monkeys, small mammals, and nearby habitats and people at the same time as measuring host infection levels		X
12. Determine role of small mammals in transmission through infection of larvae via systemic circulation and/or supporting co-feeding between nymphs and larvae: quantify burdens, age structure, feeding history, and infection rates of ticks found on live monkeys, small mammals, and nearby habitats and people		X
13. Determine whether sequence data can be used to elucidate spatial and temporal diversity in KFD, whether such diversity is linked to vector or hosts, and to infer spatial movement of KFD in order to better understand transmission and spatial scale of risk		X
Barrier: Reservoir density, distribution, habitats, and behaviour		
14. If monkeys are confirmed as important amplifying hosts for KFDV and contributing to transmission risk via infected ticks to humans, quantify their habitat associations, movement rates, and interactions with people across agroforest landscapes	X	X
15. If small mammals are confirmed as important reservoirs for KFDV and contribute to transmission to humans, quantify their habitat associations, movement rates, and interactions with people across agroforest landscapes	X	X
Barrier: Susceptibility of spillover host		
16. Investigate social and cultural barriers to uptake of the current and potential future improved vaccines across the range of affected communities in South India		
17. Test the efficacy in inducing protective immunity and assess duration of immunoprotection for the current vaccine		
18. Investigate the potential efficacy of novel vaccines and alternative vaccines such as those available for closely related viral infections		
19. Develop correlative and mechanistic predictive models of social, environmental, and ecological factors influencing spillover to better target vaccination and surveillance in the landscape		

KFD, Kyasanur Forest Disease; KFDV, Kyasanur Forest Disease Virus; WHO, World Health Organization.

Fig 1 indicates how current management recommendations for KFD in India align with the hierarchical barriers to human spillover identified in our extended framework for vector-borne diseases. From this, it is clear that current recommendations are conventional interventions largely focused on humans as the spillover host: (i) reducing exposure through community education and tick bite prevention; and (ii) reducing the number and severity of cases in humans through vaccination. Measures for reducing tick populations at perceived infection hotspots and monitoring disease events in monkeys as a sentinel vertebrate host are also encompassed in existing government KFD management recommendations.

Finally, the experiences and views of disease managers in implementing and engaging with local communities on specific current recommended management practices are summarised in Table B in S1 Appendix.

## Discussion

### Preventing tick bites on people through protective measures

Recommended protective measures, such as wearing long clothing during visits to the forest, application of tick repellents to the body and clothing, and washing the body and clothing after returning from the forest, are supported as being effective for preventing tick bites by robust empirical data from other tick-borne disease systems (refs in Table A in S1 Appendix). These measures are endorsed by health organisations such as WHO. However, there is limited empirical data for the KFD system, particularly around the effectiveness of locally available repellents, leading to the identification of several short-term research priorities (Table 3). Indeed, there is an urgent need to systematically review the natural repellents currently used by people in the Western Ghats [58] and test their efficacy compared to repellents recommended and distributed by the Indian Government (DHFWS), primarily dimethyl phthalate (DMP) oil, or those recommended by the WHO (Priority 1, Table 3). Natural repellents such as Malabar catmint *Anisomeles malabarica* have good efficacy against *Haemaphysalis bispinosa* ticks [59] but have not been tested robustly on *H*. *spinigera* and other known KFDV vectors. Numerous studies have assessed repellence of natural remedies and report variation in efficacy between vector species (reviewed in [60–62]), but comparisons between studies are hampered by a lack of robust standardised tests [60,63]. Standardised assays are needed (Priority 2), including toxicity tests (via in vivo and in vitro methods [63,64]), to assess safety and tick repellent efficacy of substances in laboratory and field conditions against tick species relevant to KFDV transmission. Such testing would require development of a bioassay facility in which pathogen-free colonies of potential tick vector species in the Western Ghats can be reared. Furthermore, rigorous experiments are needed (Priority 3) to determine whether ticks survive washing of clothes, whether such ticks pose a risk to humans if they then drop off clothes that are being dried in the environment around houses, and for what length of time clothes need to be hung up outside under different conditions before they are free of ticks and safe to be worn again.

Current management recommendations and surveillance strategies assume that people only need to protect themselves from exposure to infected ticks within forests. There is some empirical support that forests support tick species implicated in KFDV transmission [65]. However, evidence from other (sylvatic) tick-borne disease systems indicates that fragmented and ecotonal habitats at the interface with human habitation may pose a risk of infection to humans, due to such habitats supporting reservoir hosts or substantial densities of infected vectors [66–69]. Other habitats featuring in the agroforest landscape mosaic of the Western Ghats, such as fallow land, paddy, and plantation, may also pose a risk, but there is little empirical evidence on comparative densities of vectors and hosts in these habitats. A key research priority therefore (Priority 4, Table 3) is to quantify the abundance and infection rates of different tick vector species across a broad spectrum of habitats within agroforest mosaics, including human habitation, to understand how risk of exposure to infected ticks extends out from forests. Results of such empirical research in 2 affected districts in the Western Ghats is forthcoming from the MonkeyFeverRisk project of which this evaluation of management strategies is a part (https://www.monkeyfeverrisk.ceh.ac.uk/). Current tick surveillance simply targets forests [65], but stratified tick surveillance sampling and analysis by habitats and season could be integrated into the routine tick surveillance.

Overall, we recommend a new integrated approach at the local community level to prevent KFDV infection from tick bites. For example, current guidance on the use of protective clothing fails to include the need to cover up the feet and does not give guidance on the importance of regular full body checks for attached ticks or advice on how to promptly and effectively remove these ticks (Table 3). Educational leaflets and videos should be developed jointly between the health department and local communities that give guidance on best practice for preventing tick bites and removing ticks (recent community guidance material has focused on disease symptoms and vaccination and less on tick bite prevention) while also taking account of potential trade-offs between recommendations and community livelihood and health priorities (Tables A and B in S1 Appendix). Indeed, as part of our MonkeyFeverRisk project, we have codeveloped such community guidance in local languages (Kannada and Malayalam; education materials can be accessed here: https://www.monkeyfeverrisk.ceh.ac.uk/kfd).

### Preventing tick spread by cattle through protective measures

Existing management guidance recommends the application of acaricides to reduce tick loads on cattle and to prevent transportation of ticks from forests to areas of human habitation (Table A in S1 Appendix, Table 2). Cattle do support high numbers of ticks, including adult ticks and *H*. *spinigera* (the putative main vector species), although the tick species most commonly found on cattle have not yet been incriminated in the KFDV transmission cycle [44,45]. Hence, cattle may act both as an amplifier of tick numbers and as a disperser of ticks between habitats. Moreover, correlative modelling found that the spatial risk of human KFD cases was associated with the density of cattle in areas long-affected by KFD [70]. A human case–control study from the 2011 to 2012 KFD outbreak also identified handling of cattle as a significant risk factor for infection [35]. Conversely, cattle may act to dilute infection, since they do not show systemic infection with KFDV [42,43]. There is evidence from other tick-borne disease systems that increased density of ungulate hosts, which can amplify ticks but do not have systemic infection, may dilute pathogen transmission by diverting tick bites from competent hosts [71,72]. Although cattle grazing is small scale in the Western Ghats, with small numbers of cattle owned by local households (mean 2.5 per household (range 0 to 15, *n* = 229) from our MonkeyFeverRisk household survey data), there is also potential that cattle grazing may alter habitat composition and reduce or increase habitat suitability for ticks. Resolving these contrasting potential roles of cattle in KFDV transmission, by comparing the abundance, composition, and infection rates of ticks between cattle, wildlife hosts, and people, in varying ecological settings is therefore an urgent research priority (Priority 5, Table 3).

It is clear from the experiences of disease managers (Table A in S1 Appendix) that farmers cannot always exclude cattle from forests to avoid tick exposure, since the resulting shortages of fodder can lead to animal health and livelihood impacts. This highlights the imperative to identify effective personal protective measures for animals and people that cannot avoid the forests. Depending on resistance, acaricides are effective at reducing tick burdens on livestock, which may act as vectors for a range of diseases [73]. Such tick-borne infections have significant impacts on the livelihoods of poor farming communities in tropical areas by decreasing output of animal products, including manure which contributes to crop production loss [74–76]. Therefore, although the role of cattle in KFD dynamics is unclear, current management practices may prevent transmission of other significant mite- or tick-borne diseases of animals and humans which are known to be present in KFD-affected areas, including scrub typhus, Lyme disease, Indian tick typhus, babesiosis, theileriosis, and anaplasmosis [76–80]. Regardless, further testing of the effectiveness of different formulations of acaricides, including optimal doses and frequency of application, is needed, including for natural repellents that may be more readily available and commonly used within Western Ghats communities (Priority 7, Table 3). It is also essential to ascertain whether resistance against acaricides that are commonly applied both to animals and to the habitat is widespread in tick populations in India, particularly in tick species involved in KFDV transmission (see review in [81]; Priority 8).

### Avoidance of human activities

Several of the current management recommendations for KFD seek to reduce human exposure by setting out activities that should be avoided (Table A in S1 Appendix, Table 2). These include avoiding visiting forests where monkeys have died (see next section), not sitting or lying on the ground or in bushy areas of forest, and avoiding bringing dry leaves into villages. Dry leaf litter is important not only for animal bedding and fodder but also for fertilising crops where alternatives are not available [82], and the importance of this practice constituted a key theme of questions from disease managers (Table D in S1 Appendix). Although correlative modelling suggests that the presence of piles of dry leaves around the compounds of the house was associated with human disease cases [35], empirical data are needed on the abundance, infection rates, and survival of ticks found in different types of dry leaf litter, used for animal fodder and bedding, and subject to different treatments in villages (Priority 6, Table 3). More generally, the rate of contact between people and ticks during different activities in and around the forest should be quantified (Priority 9, Table 3) to obtain a clearer picture of which activities cause highest exposure ([83]; https://www.monkeyfeverrisk.ceh.ac.uk/).

### Measures to reduce density and distribution of infected ticks

The measures currently recommended to reduce the density and distribution of infected ticks include controlled burning of dry leaves and bushes in forests and around houses; dusting of insecticide (malathion) within 50 feet of a monkey carcass; and burning of monkey carcasses. There are no studies of the impacts of controlled burning regimes on potential tick vectors of KFDV in India. Evidence from other disease systems shows that burning can both reduce or increase tick abundance and infection rates and that impacts may vary with burning regimes, time, and with complex local responses of hosts, vectors, and vegetation [84,85]. Experimental investigations of the impacts of burning regimes on densities of infected nymphal ticks and their contact rates with people over short and long timescales should be conducted in the Western Ghats.

Although infected monkey carcasses may pose a risk to human health through potential direct transmission of infections via contaminated bodily fluids, burning of monkey carcasses and habitat to reduce infected tick densities are perhaps the least supported by empirical evidence among all the recommendations for KFD management and are the least likely to be effective at reducing human cases. The latter 2 measures are predicated on the assumption that humans become exposed to infected nymphal ticks mainly at hotspots around monkey deaths. Although it seems very unlikely that only the immediate area around a dead or dying infected monkey supports high density of KFDV-infected partially fed ticks or fully fed larvae (which would then feed again as nymphs after moulting) that may pass infection to humans, this should be confirmed empirically. The measures further assume that insecticide or burning will kill these partially fed ticks before they bite people (Table A in S1 Appendix). KFDV-infected monkeys are known to have high titres of virus [86] and are likely to bear infected ticks. Although partially fed nymphs have been found in areas around monkey carcasses [87] and engorged ticks have been observed to move up to 30 cm [88], no empirical data exist showing that successful interrupted feeding (intra-stadial feeding) occurs in tick species commonly found to transmit KFDV in the wild. In other tick-borne disease systems, ticks generally feed on only 1 host in each of their life stages [89]. Interrupted feeding has very rarely been recorded and most often under laboratory conditions and in *Rhipicephalus* spp. where male ticks actively seek female ticks with which to mate [90,91]. Experimental transmission studies on partially fed ticks collected from areas close to monkey deaths suggest that these have limited potential to transmit virus by feeding on a second host [87]. Thus, although dead KFDV–positive monkeys undoubtedly indicate ongoing KFDV transmission in an area, there is no direct evidence that locations of monkey deaths are hotspots of host-seeking infected ticks, compared to surrounding areas of habitat where monkeys or small mammal hosts have spent time. Moreover, evidence is building of malathion resistance in tick populations and off-target effects on human and animal health, such as increased cancer risk, elsewhere in India (Priority 8, Table 2; [92–94]).

The confusion surrounding the role that monkeys play in KFDV transmission, compared to alternative small mammal hosts, highlights the need for fundamental ecological research. Priority should be given to quantifying the tick burden on dead and dying monkeys, assessing the number, species composition, life stage, and KFDV infection rate of these attached ticks, as well as determining the number, KFDV infection rate, and blood meal identity [95] of ticks sampled from the habitat or off people near the monkey death site (Priority 10, Table 3). It also needs to be ascertained whether and how quickly partially fed ticks begin to quest, the distances they can cover, and the survival rates of non-questing partially fed nymphs and larvae that moult to the next life stage (Priority 10, Table 3).

Sampling of ticks from monkeys, small mammals, and people over a more diverse range of habitats would indicate whether monkeys are sentinels of infection or whether they contribute to KFDV transmission across broader spatial scales, by the systemic infection of large numbers of larvae or via co-feeding (transmission between larvae and nymphs feeding in close proximity; Priority 11, Table 3). Although small mammals are neglected completely in current management recommendations, given their central role in amplifying transmission in other tick-borne systems through processes such as co-feeding [96], it is equally important to clarify their role in the KFDV transmission, by sampling them in similar ways alongside monkeys and humans (Priority 12, Table 3). If small mammals and monkeys are confirmed as being important reservoirs and amplifying hosts for KFDV, then empirical data on their movements and habitat associations are required in order to quantify how far they tend to travel in the landscape (when healthy or sick for monkeys), in which habitats they interact with people and ticks, and the predictability of their movements in time and space (Priority 14 and 15, Table 3). This is vital for matching the scale of surveillance, awareness raising, and vaccination to the scale over which spillover from monkeys or small mammals occurs.

Phylodynamic approaches may be particularly promising for improving the understanding of KFD transmission. As an RNA virus, KFDV is characterised by rapid rates of molecular evolution [97,98]. Nucleotide sequence data can be used to identify genetic variation between virus population samples in time and space, infer transmission pathways, and identify dispersal and broad scale movements of pathogens between areas, offering new opportunities for elucidating the scale of KFD risk and understanding human spillover (Priority 13, Table 2; [99,100]). Moreover, assessment of the genetic diversity of KFDV strains would greatly facilitate the development of more effective vaccines (Priority 18, Table 3), as the existing vaccine, developed using virus isolates from the 1960s, may have reduced efficacy against more modern strains, as has been shown for other viruses [101].

### Measures to reduce susceptibility of people as the spillover host

A formalin-inactivated tissue culture vaccine has been used in Karnataka since 1990 [35]. The strategy of vaccinating people within a certain radius of known cases or circulation of KFDV in ticks or monkeys is well underpinned by evidence, and this vaccine is known to give protection against KFD if the correct dose procedure is followed [102,103]. However, duration of immunity from the vaccine is short, requiring multiple repeated doses, and acceptance of the vaccine and coverage rates has been poor in some areas [34,35,102], hampered by limited seasonal vaccine availability and due to social and cultural factors that need to be further investigated ([104,105]; Priority 15, Table 2). This highlights the importance and urgency in developing complementary disease prevention measures alongside vaccination that follow the new framework for disease prevention set out in Fig 1. In recent years, there is also some evidence that vaccine efficacy was reduced compared to previously [34,102], necessitating robust testing of efficacy and duration of protection offered by the current vaccine (Priority 17, Table 3) and parallel development of novel or alternative vaccines for closely related viruses which may offer cross-immunity [106] (Priority 18, Table 3).

### Surveillance in people, ticks, and monkeys to inform interventions

Finally, the current human case surveillance that underpins KFD management is well justified by empirical evidence. Human cases tend to be clustered, and surveillance of human fever cases, recommended to take place between December and May with screening for both virus (by reverse transcription PCR [RT-PCR]) and antibody presence (by ELISA), has been effective in directing vaccination campaigns [34]. Given the shifting pattern of KFD cases, with new geographical hotspots identified each year [107], we additionally recommend that fever case surveillance and serological surveys should be adopted in areas beyond the current known range of KFD cases in order to map the distribution of KFD [40] and predict disease spillover in new areas. However, serological surveys are likely to be more costly than those targeting fever cases. Targeting of human surveillance can again be guided by predictive models (Priority 19, Table 3), as is already happening in Karnataka [70] but also by detailed case–control surveys that can identify high risk livelihood groups and activities (cf. [35]).

Tick surveillance can be an effective way of predicting spillover, as demonstrated in other systems [108,109], and is valuable locally to monitor persistence of KFDV transmission over time. However, as mentioned above, tick surveillance methods could be more standardised, rigorous, and stratified by habitat (extending beyond forests) to build knowledge of tick vector habitat associations (Priority 4, Table 3) to improve targeting of subsequent interventions and surveillance. As with human surveillance, it should routinely encompass areas beyond the current front of human KFD cases. Similarly, monkey surveillance is currently implemented through passive sampling of dead and dying monkeys, and it is restricted to areas where resources are available and awareness of KFD is high (Table A in S1 Appendix). The coverage and value of this passive sampling could be extended by improving education on the importance of reporting dead and dying monkeys (perhaps providing smartphone-based app technology to facilitate such reporting), allowing standardised postmortems and tick sampling around monkey death sites to be carried out more rapidly than is current practice. Additionally, consideration could also be given to targeting small mammal maintenance hosts in future surveillance programmes. However, small mammal surveillance is likely to be time-consuming, infection duration is short lived, and high effort is needed to sample enough individuals to robustly estimate infection prevalence. Such surveillance programmes may be more achievable with improved empirical understanding of maintenance host species in order to hone selection of target species and habitats.

### Overall conclusions

Using the tick-borne KFD in India as a case study, we demonstrate how a novel barriers framework ([10,14], Fig 1) can be applied to evaluate the evidence base for current management practices for reducing zoonotic disease spillover. This approach is particularly powerful for KFD, and indeed for other neglected zoonoses such as scrub typhus and leptospirosis in India and other lower middle-income countries, where ecological and social evidence underpinning management strategies is outdated or lacking altogether. We show how this framework can be used to critically evaluate existing disease mitigation measures and focus recommendations for their improvement, identify knowledge gaps and priority areas for research, and highlight potential opportunities for new interventions.

Current management guidance for reducing the risk of human KFD primarily focuses on conventional practices to reduce human exposure and susceptibility to infection, mainly targeting the final, human barriers to spillover (Fig 1). Primary recommendations are for the use of repellents in conjunction with advice for avoiding potentially risky habitats and activities and a targeted vaccination strategy. However, our review clearly highlights the lack of robust, current empirical knowledge on the ecological and social factors leading to human cases of KFD, thus precluding the future development of a broader suite of management interventions, such as ecological interventions. This lack of empirical evidence also means that the effectiveness of current management recommendations is questionable. In particular, the role of cattle and primates in KFDV dynamics is not well understood (Table 2, Table B in S1 Appendix). Thus, management practices such as burning monkey carcasses, malathion spraying, and controlled burning of vegetation around the sites of monkey deaths are particularly unfounded. Such practices are predicated on the untested assumption that monkey deaths reflect localised hotspots of transmission, as opposed to monkeys being sentinels that indicate KFDV prevalence in the area and more widespread transmission in a range of alternative hosts. Some of the current guidance can be improved based on available evidence, and we suggest detailed ways of doing this in Table A in S1 Appendix. Improved ecological understanding of KFD disease dynamics has the potential to lead to a broader and more economical suite of potential interventions that target vector or reservoir hosts and which are more effective in the longer term at reducing human spillover [10]. Indeed, even in similarly complex, but well-studied multi-host tick-borne infections such as Lyme disease, interventions that target nonhuman hosts are possible: There is evidence that oral bait vaccines that target rodent reservoir hosts are effective at reducing the density of infected nymphs [110–112]. Entomopathogenic fungi such as *Beauveria bassiana* and *Metarhizium anisopliae* have also shown potential as biological control of tick vectors involved in Lyme disease transmission [113]. Other novel interventions are currently being developed, and much may be learned from the testing of such interventions in tick-borne disease systems where transmission dynamics are well elucidated. Vaccines are being developed, for example, against tick saliva or salivary gland proteins, in order to decrease the ability of ticks to feed on reservoir hosts, thus disrupting pathogen transmission [114–116], and self-disseminating, transmissible vaccines that are capable of spreading through wild animal reservoirs [117,118].

The new framework developed here illustrates the significant number of hierarchical barriers (Fig 1) that must be overcome in order for humans to become infected by KFDV. This further emphasises the need for a cross-disciplinary approach to provide an evidence base and implement appropriate management interventions for tick-borne diseases. Focusing research on a single barrier or barrier type within the hierarchy, within a particular scientific discipline, will not be sufficient for understanding spillover risk or implementing effective interventions [14,119]. For KFD, the ecological processes that underpin seasonal transmission dynamics, such as vector and host activity and habitat associations, seasonal resource use, and movement, need to be quantified alongside the social and cultural processes that influence ecosystem use, livelihoods, and exposure of people. For example, the majority of disease managers interviewed highlighted complex trade-offs between restricting forest access to minimise risk of exposure and safeguarding local livelihoods that need to be further investigated, as well as diverse social, cultural, and techno-administrative barriers to uptake of vaccines, tick personal protection, and surveillance measures, e.g., post mortems (Table B in S1 Appendix). Aligning with the One Health initiative, interdisciplinary approaches require collaboration across diverse disciplines including ecology, epidemiology, animal and public health, health systems, and social sciences and would enable predictive models of pathogen spillover to be refined, new interventions to be developed, and vaccination strategies and surveillance to be targeted more effectively [70].

In order to sustainably and effectively refine management interventions for neglected zoonotic diseases in the face of changing empirical knowledge and environmental and policy shifts, it is vital that strategies and research are codeveloped iteratively and reflexively across disciplines, targeting knowledge gaps and prioritising interventions identified by cross-sectoral stakeholders and involving beneficiaries alongside researchers [70,120]. Within the MonkeyFeverRisk project, empirical research and models were co-created using a coproduction approach, which placed stakeholder engagement at the heart of the research, from joint framing of the problem to knowledge integration and experimentation with resulting knowledge and tools [121–123]. In practice, active science–policy–practice interfaces between a diverse range of stakeholders, researchers, and beneficiaries were established and maintained through multi-stakeholder workshops and focus groups, inclusion of decision-makers as active research partners, researcher membership of government technical advisory committees on KFD, and through cross-sectoral WhatsApp groups setup following a direct request from stakeholders at our first workshop (https://www.monkeyfeverrisk.ceh.ac.uk/sites/default/files/Stakeholder_workshop_report_MonkeyFeverRisk_16012019.pdf). An illustration of the valuable framing of empirical research and subsequent knowledge exchange that can arise through such science–policy–practice interfaces is provided in Table D in S1 Appendix, which shows key ecological questions posed to researchers by practitioners through the 2 recent KFDV transmission seasons. The trade-offs between livelihood benefits and disease disbenefits from forests, the poor uptake of some interventions for KFD (Table B in S1 Appendix), and the wide range of traditional coping methods in use by local communities highlight the importance of meaningful involvement of local communities in the design of management strategies [123], and this is a key priority for effective management of KFD.

**Key Learning Points**

- Most zoonotic diseases have complex transmission dynamics involving communities of vector and animal hosts yet understanding of transmission is generally poor, especially for neglected tropical zoonoses.
- Better ecological understanding of transmission would facilitate the development of a broader suite of management interventions to prevent human disease spillover by strengthening barriers to transmission, including ecological interventions which rely on good knowledge of transmission ecology.
- One Health approaches that bring together disease managers and policy makers across the public health, animal health, and environmental sectors are vital for designing and implementing effective disease management.
- Kyasanur Forest Disease (KFD) is a tick-borne zoonotic disease in India and a useful model example of how frameworks identifying barriers to human spillover can be used to identify key knowledge gaps in our understanding of transmission dynamics.
- For KFD, ecological understanding of the role of small mammals, cattle, and monkeys in transmission will be critical for the development of improved management strategies.

**Top Five Papers**

1. Grace D, Gilbert J, Randolph T, Kang’ethe E. The multiple burdens of zoonotic disease and an Ecohealth approach to their assessment. Trop Anim Health Prod. 2012 Sep;44(Suppl 1):S67–73.
2. Webster JP, Gower CM, Knowles SCL, Molyneux DH, Fenton A. One health—an ecological and evolutionary framework for tackling Neglected Zoonotic Diseases. Evol Appl. 2016 Feb;9(2):313–333.
3. Sokolow SH, Nova N, Pepin KM, Peel AJ, Pulliam JRC, Manlove K, et al. Ecological interventions to prevent and manage zoonotic pathogen spillover. Philos Trans R Soc Lond B Biol Sci. 2019 Sep 30;374(1782):20180342.
4. Plowright RK, Parrish CR, McCallum H, Hudson PJ, Ko AI, Graham AL, et al. Pathways to zoonotic spillover. Nat Rev Microbiol. 2017 Aug;15(8):502–510.
5. Purse BV, Darshan N, Kasabi GS, Gerard F, Samrat A, George C, et al. Predicting disease risk areas through co-production of spatial models: The example of Kyasanur Forest Disease in India’s forest landscapes. PLoS Negl Trop Dis. 2020 Apr 7;14(4):e0008179.

## Supporting information

S1 Appendix **Table A:** Details of the current management practices recommended for preventing human cases of KFD in the Western Ghats area of India. Current management practices undertaken to prevent human cases of KFD were identified based on a number of guidance documents and sources originating from the National Centre for Disease Control and the Department of Health and Family Welfare Services: a guidance bulletin (32) and a manual of KFD (33). The management type indicates whether the measure targets reservoir hosts, vectors, or human hosts and which barrier to human spillover the management addresses (see Fig 1 in the main paper). We detail the main assumptions underpinning the management advice in terms of how such practice would reduce human transmission via infected tick bites and review the empirical support for the assumptions made. We detail responses from key informant interviews undertaken within the KFD endemic area, relating to how the current management recommendations for preventing human cases of KFD are being applied in the field in order to illustrate challenges or misconceptions associated with management practices. Finally, based on the balance of supporting empirical evidence, we recommend whether the current management practice is justified or could be improved. **Table B:** Main thematic analysis results summaries based on interviews with district and taluka managers regarding their experiences and perceptions about current KFD management in the Western Ghats area of India. **Table C:** Details of the designation of participants in the key informant interviews used to provide key quotes on the current application of management practices for KFD in the field. **Table D:** Examples of key ecological questions posed to researchers by practitioners in the 2018–2019 and 2019–2020 seasons for human KFD cases. We highlight the current knowledge gap that needs to be addressed in order to address each question and whether empirical data are currently being collected as part of the MonkeyFeverRisk project to provide evidence to fill this knowledge gap. KFD, Kyasanur Forest Disease.
(PDF)
