# Supplementary material for: Reviewing the ecological evidence base for management of emerging tropical zoonoses: Kyasanur Forest Disease in India as a case study
Source: PLoS Negl Trop Dis. 2021 Apr 1;15(4):e0009243. doi: 10.1371/journal.pntd.0009243 (PMC8016103; doi:10.1371/journal.pntd.0009243)
Supplement: S1 Appendix — Table A: Details of the current management practices recommended for preventing human cases of KFD in the Western Ghats area of India. Current management practices undertaken to prevent human cases of KFD were identified based on a number of guidance documents and sources originating from the National Centre for Disease Control and the Department of Health and Family Welfare Services: a guidance bulletin (32) and a manual of KFD (33). The management type indicates whether the measure targets reservoir hosts, vectors, or human hosts and which barrier to human spillover the management addresses (see Fig 1 in the main paper). We detail the main assumptions underpinning the management advice in terms of how such practice would reduce human transmission via infected tick bites and review the empirical support for the assumptions made. We detail responses from key informant interviews undertaken within the KFD endemic area, relating to how the current management recommendations for preventing human cases of KFD are being applied in the field in order to illustrate challenges or misconceptions associated with management practices. Finally, based on the balance of supporting empirical evidence, we recommend whether the current management practice is justified or could be improved. Table B: Main thematic analysis results summaries based on interviews with district and taluka managers regarding their experiences and perceptions about current KFD management in the Western Ghats area of India. Table C: Details of the designation of participants in the key informant interviews used to provide key quotes on the current application of management practices for KFD in the field. Table D: Examples of key ecological questions posed to researchers by practitioners in the 2018–2019 and 2019–2020 seasons for human KFD cases. We highlight the current knowledge gap that needs to be addressed in order to address each question and whether empirical data are currently being collected as part [file pntd.0009243.s001.pdf]

Reviewing the ecological evidence-base for management of emerging tropical zoonoses: Kyasanur Forest Disease in India as a case study

SJ Burthe, SM Schäfer, FA Asaaga, N Balakrishnan, MM Chanda, N Darshan, SL. Hoti, SK. Kiran, T Seshadri, PN Srinivas, AT Vanak and BV Purse

Appendix S1

Contents

**Table A:**	**Empirical support for current KFD management practices, critical evaluation of whether management is justified, and relevant responses from key informant interviews (p1)**
**Table B:**	**Summary of results on experiences and perceptions of KFD management from interview data (p16)**
**Table C:**	**Details of the participants in the key informant interviews used to provide key quotes on the current application of management practices for KFD (p17)**
**Table D:**	**Ecological questions posed to researchers by KFD practitioners, highlighting the knowledge gaps needed to address such questions (p18)**
**References**	**(p20)**

Table A: Details of the current management practices recommended for preventing human cases of KFD in the Western Ghats area of India. Current management practices undertaken to prevent human cases of KFD were identified based on a number of guidance documents and sources originating from the National Centre for Disease Control and the Department of Health and Family Welfare Services: a guidance bulletin (1), a manual of KFD (2). The management type indicates whether the measure targets reservoir hosts, vectors or human hosts and which barrier to human spillover the management addresses (see Figure 1 in the main paper). We detail the main assumptions underpinning the management advice in terms of how such practice would reduce human transmission via infected tick bites, review the empirical support for the assumptions made. We detail responses from key informant interviews undertaken within the KFD endemic area, relating to how the current management recommendations for preventing human cases of KFD are being applied in the field in order to illustrate challenges or misconceptions associated with management practices. Finally, based on the balance of supporting empirical evidence, we recommend whether the current management practice is justified or could be improved.

Reviewing the ecological evidence-base for management of emerging tropical zoonoses: Kyasanur Forest Disease in India as a case study

SJ Burthe, SM Schäfer, FA Asaaga, N Balakrishnan, MM Chanda, N Darshan, SL. Hoti, SK. Kiran, T Seshadri, PN Srinivas, AT Vanak and BV Purse

Management Type (barrier to spill-over)	Current management recommendation	Assumptions and rationale behind management guidance	Empirical evidence	Exemplar quotations from key informant interviews with disease managers on how management recommendations are currently being applied in the field	Is management justified? Can management be refined and improved
Tick bite prevention through protective measures<br><br>(Human hosts: human activities in ecosystems and spill-over host exposure to vectors)	Personal protection measures should be taken (long clothes covering neck, chest, back, and legs) before going to the forest.	Covering up the body will prevent tick bites	**Yes, this is a well-endorsed practice for tick prevention.** The World Health Organisation (WHO) recommend that clothing provides some protection if, for example, trousers are tucked into boots or socks and if shirts are tucked into trousers. However, there is strong evidence that clothes and clothing impregnated with repellents provide more protection than simply covering up (3).	"…Nobody [affected groups] wears shoes or anything and you apply the oil also, it is only going to be protective for 3 to 4 hours but these people go in the morning and come in the night, so, no oil is going to work…"	**Yes but further education** is needed as this strategy alone will not prevent tick bites. The advice needs to be updated to highlight the need to cover the feet and ankles, and that clothing needs to form a continuous protective barrier by e.g. tucking trousers into socks, shirts into trousers. We recommend an integrated approach- use of protective clothing and tick repellents, checking the entire body daily after having been in tick-infested habitats, and prompt and effective removal of any attached ticks.

Management Type (barrier to spill-over)	Current management recommendation	Assumptions and rationale behind management guidance	Empirical evidence	Exemplar quotations from key informant interviews with disease managers on how management recommendations are currently being applied in the field	Is management justified? Can management be refined and improved
Tick bite prevention through protective measures<br><br>(Human hosts: human activities in ecosystems and spill-over host exposure to vectors)	People living in the forest or visiting forest areas should use tick repellents (DMP oil, DEET, local herbs) before going to the forest. Permethrin-based repellents should be used on clothing	Applying repellents will prevent tick bites	**Yes, the use of repellents is well established and endorsed.** The WHO recommend effective repellents that prevent ticks from attaching to the body include DEET, dimethyl phthalate, benzyl benzoate, dimethyl carbamate and indalone (3); and the U.S. Centres for Disease Control and Prevention recommend permethrin based repellents on clothing and ≥20% DEET on skin (4). Repellents may last longer if applied to clothing. However, both permethrin and DEET-based repellents are not be widely available in India and are prohibitively expensive. Local repellents vary in efficacy and there is currently no local guidance on when and how often to apply. DMP oil is distributed in areas where KFDV has previously been reported but is not very effective (5). Use of natural repellents has been reported in some areas of the Western Ghats (6) but efficacy is unknown. Repellents do not last more than a few hours when applied directly to the skin due to sweating, absorption and abrasion.	"…We ask them to apply DMP oil whenever they go to forest; I think few people… hardly 20% follow the precautions we suggest during outbreaks."<br><br>"…Nobody [affected groups] wears shoes or anything and you apply the oil also, it is only going to be protective for 3 to 4 hours but these people go in the morning and come in the night, so, no oil is going to work…"<br><br>"We are also trying to create habits on that but why should we do pressurise so much on them, we know that it is tick repellent and even mosquito repellent…, it is good if it is used regularly, even if you say all this, they don't use it. So, we have not understood why that gap still exists."	**Yes but further education is needed** as this strategy alone will not prevent tick bites and the provided repellents are not very effective (DMP oil). We recommend an integrated approach- use of protective clothing and tick repellents, checking the entire body daily after having been in risky habitats, and prompt and effective removal of any attached ticks. Although there is evidence that forests have high tick densities, there is risk of tick-bites in other habitats too and this needs to be made clear.

Management Type (barrier to spill-over)	Current management recommendation	Assumptions and rationale behind management guidance	Empirical evidence	Exemplar quotations from key informant interviews with disease managers on how management recommendations are currently being applied in the field	Is management justified? Can management be refined and improved
Tick bite prevention through protective measures<br><br>(Human hosts: human activities in ecosystems and spill-over host exposure to vectors)	People should wash their clothes and body with hot water and soap after returning from the forest.	Washing clothes and body will remove ticks	Yes, the removal and washing of potentially contaminated clothes is a well endorsed recommendation. The WHO recommends that clothing should be removed and examined for the presence of ticks after a tick-infested area has been visited (3). However, there is evidence that ticks can survive even after washing clothes using washing machines and dryers (7,8). Most households in rural India hand-wash clothes in cold water, which will be ineffective at removing ticks. Washing the body may remove unattached ticks but will not remove attached ones. Moreover, current guidance is misleading because it leads to the widespread belief that taking a shower will remove ticks and hence reduce the need for other protective measures such as daily body checks, or use of repellents.		Yes regarding washing of clothes but this will not be completely effective. Need to also warn people not to bring potentially contaminated clothes inside their homes and to have clear recommendations on how long clothes should be hung up outside to ensure they are tick-free. Is important to quantify how far any surviving ticks are able to move if they drop off contaminated laundry. There is a need to educate people in how to check their bodies for ticks and safely remove them after having entered tick-infested habitats, and to warn them that showering will not remove attached ticks. Health centres recommended to distribute tools for safe removal of ticks.

Management Type (barrier to spill-over)	Current management recommendation	Assumptions and rationale behind management guidance	Empirical evidence	Exemplar quotations from key informant interviews with disease managers on how management recommendations are currently being applied in the field	Is management justified? Can management be refined and improved
Reduction of ticks in habitat<br><br>(vectors: vector host associations and contact rates with reservoirs)	The application of insecticide (Malathion) may be carried out in areas where monkey deaths have been reported within a radius of 50 feet around the spot of the monkey death. It is also effective on forest tracks frequently visited by people for various activities.	Assumes that the main risk for humans in being bitten by infected ticks arises at a hotspot where ticks may leave a dying or dead monkey with high KFDV viraemia. The purpose of insecticide application is to kill both partially fed and fully fed ticks (which may moult into infected nymph).	KFDV-infected monkeys are known to have high titres of virus (9) and so are likely to bear infected ticks. There is evidence that engorged ticks can move up to 30cm (10) but no empirical data exists on whether and how successfully interrupted feeding occurs in the species of tick most commonly found to transmit KFDV.<br>Partially-fed ticks infected with KFDV have been found at sites of monkey-deaths, but experimental transmission studies suggest that these have limited potential to transmit virus by feeding on a second host (11). Indeed, such interrupted feeding (intra-stadial feeding) has only rarely been recorded in other tick-borne disease systems and most often under laboratory conditions, for example in *Rhipicephalus* spp. (12,13). There is therefore no direct empirical evidence that locations of monkey deaths are hotspots of host-seeking infected ticks capable of transmitting the virus to humans.		Intervention is not currently justified, further empirical evidence is needed to address whether this is a valid management practice. Humans are mainly infected by nymphal ticks (14). For the dead monkey to constitute an infection risk to humans, either infected partially-fed nymphs must be leaving the dead monkey and searching for a new host (which assumes that intra-stadial feeding is occurring) or infected larvae, infected either by co-feeding or systemically, leave the dead monkey and then pose a risk to humans after they have moulted to become nymphs and seek new hosts. Management currently assumes that the focal risk arises from ticks leaving the dead monkey- if no questing behaviour is occurring in partially-fed nymphs then the likely rate of potentially infected and viable ticks arising from the host is no greater than for any suitable habitat where the monkey has spent time. Additionally, there is evidence from tick populations in other parts of India that malathion resistance can be an issue in tick control and also that malathion can have implications for human and animal health, such as limited evidence it can increase cancer risk (15).

Management Type (barrier to spill-over)	Current management recommendation	Assumptions and rationale behind management guidance	Empirical evidence	Exemplar quotations from key informant interviews with disease managers on how management recommendations are currently being applied in the field	Is management justified? Can management be refined and improved
Prevention of tick bites on livestock<br><br>(vectors: vector density, distributions, habitats and behaviour)	Application of insecticide on cattle can prevent transportation of ticks from forests to dwelling premises.	Cattle have high tick loads and are capable of moving infected ticks from high-risk habitats such as forests into the vicinity of human habitation. Tick loads on cattle will be reduced by the application of appropriate acaricides.	Cattle are known to have high tick loads, including high loads of feeding adults and including *H. spinigera*, although the most common tick species found on cattle have not been incriminated in the KFDV transmission cycle (16,17). Hence, cattle may act both as an amplifier of tick numbers and as a disperser of ticks between habitats.<br><br>Handling of cattle was also identified as a significant risk factor associated with human KFD infection in a case-control study from the 2011-12 outbreak (18).<br><br>However, conversely cattle may act to dilute KFDV infection. Cattle themselves do not show systemic infection with KFDV (19). There is evidence from other systems that increased density of ungulate hosts which can amplify ticks but don't have systemic infection, may dilute pathogen transmission by diverting tick bites from competent hosts (20,21).<br><br>Acaricides are used widely globally to reduce tick loads on cattle, however, effectiveness depends on the substance used, the species of ticks being targeted and on whether there is acaricide-resistance in tick populations (see review by (22)).	"…When pet animals visit the forest, not only buffalo, rat and dog also, they have a chance to carry ticks on their body, those nymph stage ticks become adult. In this situation, veterinary department helps to control the adult tick by applying chemical [acaricides] on the animal body."<br><br>"We said to people that do not leave the cattle to the forest because they carry ticks to home so that leads to disease. Though it [KFDV] does not come from adult ticks still that is a carrier, that lie egg, that multiply and increase in their count. Fodder shortage problem happen because animals did not leave to the forest."<br><br>"See, now animals go to forest for grazing and comes back to home, for that anti tick measures should be taken because ticks might come from cattle also…"	No, because it is currently unknown whether cattle may be acting as tick (and indirectly disease) amplifiers or whether they are acting to dilute infection risk. Cattle may be moving infected ticks to human habitations. Cattle have high tick infestation levels (particularly adults, with fully fed females capable of laying thousands of eggs and giving rise to hotspots of larval ticks) and large activity ranges. They may be maintaining high tick densities in habitats around villages, leading to higher vector availability for hosts that can potentially transmit KFDV.<br><br>Although acaricides can reduce tick burdens on cattle, there may be issues with resistance to these pesticides (22). There is also a need for clear guidance on the choice of appropriate acaricide and/or repellent for livestock, and frequency of application.

Management Type (barrier to spill-over)	Current management recommendation	Assumptions and rationale behind management guidance	Empirical evidence	Exemplar quotations from key informant interviews with disease managers on how management recommendations are currently being applied in the field	Is management justified? Can management be refined and improved
Reduction of ticks in habitat<br><br>(vectors: vector density, distributions, habitats and behaviour)	Controlled burning of the dry leaves and bushes in the forest boundaries, premises of human habitats.	Assumes burning of vegetation will reduce tick densities and prevent human transmission of KFDV.	Evidence on whether burning reduces tick densities in other systems is mixed. In general, there is evidence that repeated burning can reduce tick densities in the long-term. For example, (23) found that repeated burning does not reduce pathogen prevalence but did reduce tick encounter rates and thus lower risk of pathogen transmission in the USA. However, vector and host responses to fire may be complex and difficult to predict. A study of natural wildfires in California found that questing tick densities were higher in the year following the fire with declines in subsequent years (24). There is a risk that burning may increase tick density in the short-term, or could potentially lead to altered host movements that may increase infection risk.		Management is not justified as is not supported by empirical evidence, with no robust studies of the impacts of burning regimes on tick species implicated in KFDV transmission having been undertaken in India. The outcomes of prescribed burning can be difficult to predict and could potentially increase human infection risk in the short-term. Burning may also alter vegetation structure and proportion of invasive plant species, and has been used in some areas to remove local forest and make areas easier to convert to agricultural land, potentially increasing tick densities in the longer-term. Moreover, it has been suggested that increased frequency of fires represents a persistent conservation threat to forest ecosystems in the Western Ghats (25)

Management Type (barrier to spill-over)	Current management recommendation	Assumptions and rationale behind management guidance	Empirical evidence	Exemplar quotations from key informant interviews with disease managers on how management recommendations are currently being applied in the field	Is management justified? Can management be refined and improved
Reduction of ticks in habitat<br><br>(vectors: vector density, distributions, habitats and behaviour)	Burning of monkey carcass	Assumes that the main risk for humans being bitten by infected ticks arises at a hotspot where ticks may leave a dying or dead monkey with high viraemia for KFDV and that burning of the monkey carcass will kill and thus prevent ticks from leaving the host. Also reduces the likelihood for transmission of other potential pathogens via contamination from bodily fluids.	Monkeys are known to have high titres of virus and so are likely to have infected ticks and systemic infection (see above).		Yes, controlled burning is an effective way of removing a potentially infected carcass. However, there is a need for robust, prompt and effective post-mortem studies on freshly dead monkeys in order to confirm infection status and to collect ticks in order to address empirical knowledge gaps (see above), so co-ordination between health and forest departments, surveillance teams and locals is necessary to establish best practice.

Vaccination<br><br>(Human hosts: susceptibility and infection)	Vaccination is conducted within a 5km radius of an area which has reported either a positive human or monkey case of KFD or a positive KFDV-infected tick in the past 5 years. It is discontinued if that area has not reported any KFDV positivity for the past 5 years	Vaccination of people in areas where KFD is known to be a risk will prevent human cases of the disease.	Yes. The vaccine is known to give protection against KFD if the correct dose procedure is followed (26,27). However, in recent outbreaks there is some evidence that vaccine efficacy was reduced compared to previous outbreaks and there have been problems with poor uptake of the vaccine in some areas (27,28).	"…And vaccine, first dose immunity is only 33% of immunity, second dose you get around 60%, booster dose you get around 80, after 5 doses, 5 years, you get about 90% immunity."<br><br>"As far as KFD is concerned, no definite proper research has happened. We are struggling with the age-old vaccine, which was prepared in the 90s I think. We are going with the same. We don't know about the strain change…the virus... Even the research has not done. Even the cases which (who were) vaccinated fully, also were [re]infected. So, for that we need to do some research on whether the prevalence has changed it or not."<br><br>"Only problem is with, in my view, the vaccine… vaccine is the main hitches. Because the acceptance of that vaccine is not so… welcoming sign is not seen." One more point is the doses also. We need to give multiple doses to get what we need. To get some protection he/she needs to take full course. After that every year he/she needs to get booster dose for five years. Those are all hitches. I think one single injection that can protect the person for five years is needed. If we invent such vaccines then we can contain these measures, in my view."<br>"..Where the vaccination is good, there should be less number of cases… but still we are seeing cases so… There that time we had a doubt whether the vaccination is working properly but I think it is not about the vaccine only – the timing of the vaccine is also important – like they have to take the vaccine at the specified time otherwise the potency of the vaccine would be decreased."	Yes but targeting of areas could be improved beyond responding to outbreaks. Modelling ecological and social factors linked to human disease cases and barriers to vaccine uptake can provide more tailored risk maps and help target vaccination strategies.

Reviewing the ecological evidence-base for management of emerging tropical zoonoses: Kyasanur Forest Disease in India as a case study

SJ Burthe, SM Schäfer, FA Asaaga, N Balakrishnan, MM Chanda, N Darshan, SL. Hoti, SK. Kiran, T Seshadri, PN Srinivas, AT Vanak and BV Purse

Management Type (barrier to spill-over)	Current management recommendation	Assumptions and rationale behind management guidance	Empirical evidence	Exemplar quotations from key informant interviews with disease managers on how management recommendations are currently being applied in the field	Is management justified? Can management be refined and improved
Education<br><br>(Human hosts: human activities in ecosystems and spill-over host exposure to vectors)	Educate villagers to avoid the forests areas where monkeys have died. Don't visit the area where recent monkey death has been reported, especially an area where case of KFD has occurred in the past.	This assumes that forests are the most risky habitats for ticks, particularly if they have been the location of monkey deaths. Assumes that the main risk for humans being bitten by infected ticks arises at a hotspot where ticks may leave a dying or dead monkey with high KFDV viraemia.	There is modelling evidence that forests may have high tick densities relative to other habitats (29) and evidence that human disease emergence was associated with deforestation (30), but robust empirical systematic studies of tick habitat associations across a broad suite of habitat classes are lacking for the Western Ghats.	"Sir, advising people to not go to forest is the most difficult part. Because their life is dependent on going to the forest, so we are unable to prevent or change that behaviour.<br><br>"Awareness about KFD, they were aware there is monkey disease, but very less people were aware that this disease comes from tick bite. That kind of awareness, even when we surveyed in 2013 and 14, it was around only 48% or 52% awareness that it is transferred by tick-bite. Otherwise others were telling like it [KFDV] transmits by mosquito bite, it comes from water, air and others."	The current recommendation implies that forests are only risky if monkey deaths are known to have occurred. There is a need for further clarification to educate people that forests may be risky habitats with or without monkey deaths due to the high suitability of forests for supporting dense tick populations.<br><br>Recommendations to avoid forests need to be balanced with the fact that people depend on the forests for their livelihoods and animal health through the provision of animal fodder, fertiliser and other forest products. Thus, there is a requirement to find effective personal protective measures for humans and to understand the role of livestock in the KFDV cycle.

Management Type (barrier to spill-over)	Current management recommendation	Assumptions and rationale behind management guidance	Empirical evidence	Exemplar quotations from key informant interviews with disease managers on how management recommendations are currently being applied in the field	Is management justified? Can management be refined and improved
Reduce exposure through avoidance of human activities<br><br>(Human hosts: human activities in ecosystems and spill-over host exposure to vectors)	Don't bring the leaves of trees from KFD infected area to the village for cattle bedding material.	This assumes that forests are the most risky habitats for ticks, and that gathering of leaves is a risky activity. Risks arises through risk of tick bites from forest vegetation during the leaf gathering process, and also because the leaves themselves can harbour tick populations, leading to increased risk of transferring infected ticks around the home.	See above. No empirical evidence testing whether ticks can survive in leaf litter for a long enough period to be brought back to the home and reach susceptible hosts.<br><br>Having piles of leaves close to dwelling places was identified as a significant risk factor associated with human KFD infection in a case-control study from the 2011-12 outbreak (18).	"Whatever you tell like don't bring dry leaves, fodder, plants and produce from forest… I think few people as few follow the precautions we suggest during outbreaks."<br><br>"Sir, advising people to not go to forest is the most difficult part. Because their life is dependent on going to the forest, so we are unable to prevent or change that behaviour.	Yes, because there is some empirical evidence that leaf litter used as bedding in cattle barns can harbour ticks (MonkeyFeverRisk project, *Unpublished data*). There is a need for robust systematic empirical evidence assessing whether forests have the highest prevalence of ticks infected with KFDV (and see above).<br><br>Such recommendations need to be balanced with the fact that people may depend on the use of such leaves for services such as animal bedding, fertiliser and fodder, and that alternative sources may not be available. Thus, there is a requirement to find effective personal protective measures for humans and to understand the role of livestock in the KFDV cycle.

Management Type (barrier to spill-over)	Current management recommendation	Assumptions and rationale behind management guidance	Empirical evidence	Exemplar quotations from key informant interviews with disease managers on how management recommendations are currently being applied in the field	Is management justified? Can management be refined and improved
Tick bite prevention<br><br>(Human hosts: human activities in ecosystems and spill-over host exposure to vectors)	Don't handle the infected monkey carcass by bare hand without personal protective equipment.	Assumes that monkey carcasses are a potential source of infected ticks that may transmit KFDV to human hosts during the handling of monkey carcasses. Also assumes that there may be potential infection via contamination from bodily fluids.	Monkeys are known to have high titres of KFDV virus and so are likely to have infected ticks and systemic infection (see above). Risk of handling infected bodily fluids is unknown.	"…Even while burning monkey carcass, they handle with bare hands without proper PPE and precautions. They also won't be having precautions…"<br><br>"…There used to be one group D, health inspector and medical officer and together they were supposed to do it. This was from the KFD unit…Before that we used to close with a local plastic sheet until they come, and after they come, they used to perform autopsy in the open and take it and burn it. With bare hands!"	Yes, although the likelihood of intra-stadial transmission seems unlikely (see above), monkeys can have high titres of virus and there is a risk of transmission via inhalation of aerosols or direct transmission via infected blood. Human cases of KFD in laboratory workers infected via inhalation of aerosols has been reported (31). Moreover, monkeys potentially harbour other zoonotic infections, and adopting effective personal protection is justified and appropriate.
Reduce exposure through avoidance of human activities through education<br><br>(Human hosts: human activities in ecosystems and spill-over host exposure to vectors)	Highlighting risky activities: for example to not sit on the ground or in bushy areas of the forest	Assumes that particular habitats (forests) and particular activities carry a higher risk of getting tick bites	Yes. Risk of getting bitten by ticks will increase with time spent in habitats with high tick densities and activities which expose humans to ticks. However, there is currently a lack of robust empirical assessment of how social factors such as livelihoods and behaviour influence infection and tick-bite rates.	"Difficulties, they don't follow strictly whatever we say and we know that, it is difficult to follow because if we advise them not to go to forest, it is not possible as their livelihood depends on it…"<br><br>"…One is the gap between acceptance of our services offered by health department and acceptance level of the beneficiaries, there is big gap, they are not ready to accept us, we are not able to make it out why it is so."	Yes, although a more integrated approach is needed. People need educating to make them aware of risky habitats and behaviours, and why it is important to use protective clothing, repellents and undertake prompt removal of any attached ticks.

Management Type (barrier to spill-over)	Current management recommendation	Assumptions and rationale behind management guidance	Empirical evidence	Exemplar quotations from key informant interviews with disease managers on how management recommendations are currently being applied in the field	Is management justified? Can management be refined and improved
Surveillance<br><br>(Human hosts: susceptibility and infection)	Human disease surveillance of fever cases (November to June) with sera screened for KFDV	Assumes that human cases are likely to be clustered in the environment and hence that screening may allow targeted prevention strategies such as vaccination	Yes, human cases tend to be clustered and identification of cases has been used to target vaccination (28).	"…The handicap thing is, this season, so, it is rainy or it is very cold or there is no road, there is no vehicle to go and one house you visit and come back, it takes one day. So, the whole team, manpower is wasted to save one person, the one house, the other person affected and die and you can't identify them…"	Yes, this is justified.
Surveillance<br><br>(potential to inform multiple barriers)	Tick surveillance- surveillance is undertaken within 5km of areas where human cases were recorded in the previous year (for up to five years) or within 5km of areas with current monkey deaths. Surveillance is not undertaken if current human cases are recorded.	Ticks are the known vectors for KFDV and hence surveillance of infection levels is useful for predicting the severity and locations of outbreaks.	Yes, many species of ticks, primarily *Haemaphysalis* species, are known to be vectors for KFDV and surveillance is used to predict spill-over in other systems (32,33).	"Mainly the lack of trained and experienced entomologists is the challenge. There is only one person (for the district possibly), for some people training has been given and there is a need to supervise them. So, they tell us to train to ASHA (health) workers and male health worker, but that is risky now. How can they with minimum training and precautions do tick surveillance? That was not their original training or their role.<br><br>"Even if the surveillance is there, the quality should be important. So, if tick surveillance is there then they (health workers) have to be trained properly. They don't know which species – so that also they have to be trained for different aspects of tick collection like what are the different methods that can be done and what are the better methods for collection of ticks. It depends on the landscape that is there because different landscapes require different methods for collection I feel."	Yes, tick surveillance is important and justified. Identifying areas with infected ticks would facilitate responses such as vaccination drives. However, current surveillance does not identify ticks to species level, reducing the effectiveness of surveillance at identifying vector hosts and habitat associations responsible for spill-over risk

Surveillance<br><br>(potential to inform multiple barriers)	Monkey disease surveillance	Currently, monkeys are thought to be important in the transmission cycle of KFDV to humans and therefore surveillance of monkey deaths is important.	It is currently unclear, due to a lack of robust empirical data, whether monkeys are involved in the transmission of KFD to humans or whether they act as sentinels for high prevalence of infection in ticks that may have been infected from other hosts.	"…Lack of prompt reporting with monkey death occurring outside the forest reserves going unnoticed."<br><br>"First is monkey deaths are not immediately reported so there is a chance of KFD spreading there. So, focus is not known first. Even in Aralagudu what happened is we never had monkey deaths reported. Actually, they had monkey deaths but none of them were reported. So, we didn't know there was a focus of KFD over there. Until unless the cases started appearing. That is very important I feel that the monkey deaths need to be reported immediately.<br><br>"As per norm, we are not going to conduct the post mortem in that PHC area if suddenly positive case reports. Once positive report in human, ticks, or monkey within 5km PHC area, there is no question of repeated post-mortem because we have to dispose the carcasses as early as possible. Important is health and forest, who burn it and health department spread the malathion."<br>"Staff shortage affecting surveillance efforts."<br>"It is a challenge. If, there is a monkey death, they (the vets or forest department or others) tell some other reason and burn it and dispose it but every monkey death should go to post mortem examination for cause of death and tissue analysis should be done and should be followed up for whether it is KFD or not…"<br><br>"…Our group consist of 10 members in this taluk. If monkey death reported within 12	Yes, regardless of the role of monkeys in transmission of KFDV to humans, they are susceptible to KFDV and may indicate risk of human infection in an area.

Management Type (barrier to spill-over)	Current management recommendation	Assumptions and rationale behind management guidance	Empirical evidence	Exemplar quotations from key informant interviews with disease managers on how management recommendations are currently being applied in the field	Is management justified? Can management be refined and improved
				hour of death, we used to reach the spot within 2 to 3 hour, conducting the post mortem and used to do sample collection. Our role was very important in disease diagnosis in term of monkey death."<br><br>"…Even in taluka level also, coordination meeting is happening from last year [2018]. So, from last year there is little coordination but still in my opinion, it should go to down, not only taluka level, if it goes to PHC [primary health centre] level and if it works, there everyone like forest guard comes, veterinary helpers will come, local PDOs should come, then better activities will happen I think."<br><br>"In future, if we cannot control monkey, we cannot control ticks..."	

Table B: Main thematic analysis results summaries based on interviews with district and taluka managers regarding their experiences and perceptions about current KFD management in the Western Ghats area of India.

Main Themes	Sub-themes and frequency cited
I. Human activities in ecosystems	i. Complex trade-off between restricting forest access to minimise risk of exposure and safeguarding local livelihoods (9 out 11 interviewees)
II. Prevention of tick-bites on people through of personal protection measures	i. Limited usage of DMP oil and uptake of other recommended personal protection measures (11 out of 10 interviewees)
III.Social and cultural barriers to uptake of current and future potential vaccine technologies	i. Pain and discomfort concerns with existing vaccine (11 out of 11 interviewees)
	ii.Underlying religio-cultural sentiments and practices (7 out of 11 interviewees)
	iii. Anxiety caused by lack of knowledge about KFD and its transmission pathways (7 out of 11 interviewees)
	iv.Trust and legitimacy concerns (8 out of 11 interviewees)
IV.Techno-administrative barriers to uptake of current vaccine and improvement considerations	i. Vaccination coverage and availability (4 out of 11 interviewees)
	ii. Concerns about the efficacy of existing vaccine(7 out of 11 interviewees)
V. Inter-sectoral action for KFD surveillance and management	i. Increasing (district level) inter-departmental coordination during and post-outbreak situations (9 out of 11 interviewees)
	ii. Increasing policy and media attention on KFD and attendant issues (3 out of 11 interviewees)
	iii.Staffing, infrastructural and logistical challenges hampering effective coordination (6 out of 11 interviewees)
	iv. Training and capacity building of personnel in tick surveillance (6 out of 11 interviewees)

Table C: Details of the designation of participants in the key informant interviews used to provide key quotes on the current application of management practices for KFD in the field.

**Department**	**Designation of participants**	**Number of participants**	**Level of operation** (District /Taluk/ Local)
Animal Husbandry	District officers	2	District
	Animal health services manager	1	Taluk
	Taluk official	1	Taluk
Health & Family Welfare	District health officials	5	District
	Senior health worker	1	Taluk
	Medical officer	1	Local
Total		11	

Table D: Examples of key ecological questions posed to researchers by practitioners in the 2018-19 and 2019-20 seasons for human KFD cases. We highlight the current knowledge gap that needs to be addressed in order to address each question and whether empirical data are currently being collected as part of the MonkeyFeverRisk project to provide evidence to fill this knowledge gap.

**Key Questions identified via Whatsapp**	**Research that is needed to answer question and address knowledge gap**	**Details of relevant data collection from MonkeyFeverRisk project**
Dry leaves are transported from forest areas to be used as crop fertilizer 12 km distant: is there any evidence that such leaf litter harbours ticks and what alternatives would be advised?<br><br>Dry leaves is a organic fertilizer villagers use, what alternative you advise. Is there any scientific work on this fertilizer having ticks? Villagers are already under lots of stress. If you really has done tick collection from this fertilizer it's okay	(Need to) quantify abundance and infection rates of ticks found in different types of dry leaf litter used for animal fodder and bedding, under different treatments in villages (Research Priority 6, Table 2).	Tick sampling (by dragging and flagging) was undertaken from leaf litter collected as fodder, animal bedding and fertilizer for both fresh leaf litter and leaf litter that had been stored for varying lengths of time. Taxonomic identification and assessment of KFDV infection of tick samples is currently being undertaken.
A monkey sanctuary was planned to be set up within the Shimogga district, within endemic KFD area, to deal with problem monkeys (destroying crops and buildings): what if monkeys were infected with KFDV? What adverse effects could the monkey sanctuary have on other primates in the area?	Determine the role of dead and dying monkeys in generating hotspots of transmission. Need to determine role of live monkeys in KFDV transmission through infection of larvae via systemic circulation and/or supporting co-feeding between nymphs and larvae: quantify burdens, age structure, feeding history, and infection rates of ticks found on live monkeys, small mammals, and nearby habitats and people at the same time as measuring host infection levels. If monkeys are confirmed as important amplifying hosts for KFDV and contributing to transmission to humans, quantify their habitat associations, movement rates and interactions with people across agro-forest landscapes (Research Priorities 10, 11 and 13, Table 2).	Ticks were sampled in a robust, stratified way across habitats, including areas close to the sites of monkey deaths in order to ascertain whether monkeys represent hot-spots of infection risk or whether they may be acting as sentinels of risk across a broader area of habitat. Laboratory processing of samples (species identification and KFDV-testing of ticks) is currently being undertaken.

**Key Questions identified via Whatsapp**	**Research that is needed to answer question and address knowledge gap**	**Details of relevant data collection from MonkeyFeverRisk project**
Guidelines for Malathion dusting	Determine the role of dead and dying monkeys in generating hotspots of transmission. Need to determine role of live monkeys in KFDV transmission through infection of larvae via systemic circulation and/or supporting co-feeding between nymphs and larvae: quantify burdens, age structure, feeding history, and infection rates of ticks found on live monkeys, small mammals, and nearby habitats and people at the same time as measuring host infection levels. If monkeys are confirmed as important amplifying hosts for KFDV and contributing to transmission to humans, quantify their habitat associations, movement rates and interactions with people across agro-forest landscapes (Research Priorities 10, 11 and 13, Table 2).	We did not produce guidelines for this. Current management guidelines stipulate that malathion dusting should be undertaken within 50 feet of an area where a monkey has died. However, this is predicated on the assumption that the area close to monkey deaths is the main focus of risk from infected tick bites, whereas it is possible that the scale of risk is broader. In order to address this ticks were sampled in a robust, stratified way across habitats, including areas close to the sites of monkey deaths in order to ascertain whether monkeys represent hot-spots of infection risk or whether they may be acting as sentinels of risk across a broader area of habitat. Laboratory processing of samples (species identification and KFDV-testing of ticks) is currently being undertaken.
Request for certain Standard Operating Procedures from the stakeholders, for clinical management of KFD, for outbreak investigation, for monkey autopsy, for drag and flag for tick surveillance were requested.	NA	We have developed videos illustrating good practice for sampling ticks from the environment via dragging and flagging in order to provide guidance for tick surveillance. It is imperative to engage with stakeholders in order to establish the purposes of surveillance before devising clear protocols and surveillance strategies. For example, if the purpose of surveillance is to maximise the chances of finding an infected tick then should focus sampling within habitats with the greatest density of infected ticks. If the purpose is to better understand scale of risk and better understand disease-vector-habitat associations then need stratified sampling across habitats and across a broader spatial scale than at the village level.

Bibliography

1. National Centre for Disease Control. Kyasanur Forest Disease: a public health concern. 2019 [cited 2020 Feb 25]; Available from: https://idsp.nic.in/WriteReadData/l892s/60398414361527247979.pdf

2. Directorate of Health and Family Welfare Services, Govt of Karnataka. Manual on Kyasanur Forest Disease. Directorate of Health and Family Welfare Services, Govt of Karnataka; 2005.

3. World Health Organisation. Vector Control [Internet]. 1997 [cited 2020 Mar 4]. Available from: https://www.who.int/docstore/water_sanitation_health/vectcontrol/ch26.htm

4. Centers for Disease Control and Prevention. CDC yellow book 2020: health information for international travel. Brunette GW, Nemhauser JB, editors. Oxford University Press; 2019.

5. Kumar S, Prakash S, Kaushik MP, Rao KM. Comparative activity of three repellents against the ticks Rhipicephalus sanguineus and Argas persicus. Med Vet Entomol. 1992 Jan;6(1):47–50.

6. Venkatachalapathi A, Sangeeth T, Ali MA, Tamilselvi SS, Paulsamy S. Ethnomedicinal assessment of Irula tribes of Walayar valley of Southern Western Ghats, India. Saudi J Biol Sci. 2016 Oct;

7. Nelson CA, Hayes CM, Markowitz MA, Flynn JJ, Graham AC, Delorey MJ, et al. The heat is on: Killing blacklegged ticks in residential washers and dryers to prevent tickborne diseases. Ticks Tick Borne Dis. 2016 Apr 28;7(5):958–963.

8. Carroll JF. A cautionary note: survival of nymphs of two species of ticks (Acari: Ixodidae) among clothes laundered in an automatic washer. J Med Entomol. 2003 Sep;40(5):732–736.

9. Shah KV, Dandawate CN, Bhatt PN. Kyasanur forest disease virus: viremia and challenge studies in monkeys with evidence of cross-protection by Langat virus infection. [version 1; peer review: 3 approved]. F1000Res. 2012 Dec 7;1:61.

10. Bhat HR. Observations on the biology of Haemaphysalis spinigera Neumann, 1897 (Acarina: Ixodidae) under Natural conditions in KFD area. . Journal of Bombay Natural History Societ. 1986;82:548–562.

11. Sreenivasan MA, Bhat HR, Rajagopalan PK. Studies on the transmission of Kyasanur forest disease virus by partly fed ixodid ticks. Indian J Med Res. 1979 May;69:708–713.

12. Wang H, Nuttall PA. Intra-stadial tick-borne Thogoto virus (Orthomyxoviridae) transmission: accelerated arbovirus transmission triggered by host death. Parasitology. 2001 Apr;122(Pt 4):439–446.

13. Little SE, Hostetler J, Kocan KM. Movement of Rhipicephalus sanguineus adults between co-housed dogs during active feeding. Vet Parasitol. 2007 Nov 30;150(1-2):139–145.

14. Virus Research Centre, Pune, India. Kyasanur Forest Disease 1957-1964. Indian Council of Medical Research; 1964.

15. Brasil VLM, Ramos Pinto MB, Bonan RF, Kowalski LP, da Cruz Perez DE. Pesticides as risk factors for head and neck cancer: A review. J Oral Pathol Med. 2018 Aug;47(7):641–651.

16. Rajagopalan PK, Patil AP, Boshell J. Ixodid ticks on their mammalian hosts in the Kyasanur Forest disease area of Mysore State, India, 1961-64. Indian J Med Res. 1968 Apr;56(4):510–525.

17. Balasubramanian R, Yadav PD, Sahina S, Arathy Nadh V. Distribution and prevalence of ticks on livestock population in endemic area of Kyasanur forest disease in Western Ghats of Kerala, South India. J Parasit Dis. 2019 Jun;43(2):256–262.

18. Kasabi GS, Murhekar MV, Yadav PD, Raghunandan R, Kiran SK, Sandhya VK, et al. Kyasanur Forest disease, India, 2011-2012. Emerging Infect Dis. 2013 Feb;19(2):278–281.

19. Anderson CR, Singh KR. The reaction of cattle to Kyasanur Forest disease virus. Indian J Med Res. 1971 Feb;59(2):195–198.

20. Bolzoni L, Rosà R, Cagnacci F, Rizzoli A. Effect of deer density on tick infestation of rodents and the hazard of tick-borne encephalitis. II: population and infection models. Int J Parasitol. 2012 Apr;42(4):373–381.

21. Levi T, Keesing F, Holt RD, Barfield M, Ostfeld RS. Quantifying dilution and amplification in a community of hosts for tick-borne pathogens. Ecol Appl. 2016 Mar;26(2):484–498.

22. George JE, Pound JM, Davey RB. Chemical control of ticks on cattle and the resistance of these parasites to acaricides. Parasitology. 2004 Oct;129(7):S353–S366.

23. Gleim ER, Conner LM, Berghaus RD, Levin ML, Zemtsova GE, Yabsley MJ. The phenology of ticks and the effects of long-term prescribed burning on tick population dynamics in southwestern Georgia and northwestern Florida. PLoS One. 2014 Nov 6;9(11):e112174.

24. MacDonald AJ, Hyon DW, McDaniels A. Risk of vector tick exposure initially increases, then declines through time in response to wildfire in California. 2018;

25. Kodandapani N, Cochrane MA, Sukumar R. Conservation threat of increasing fire frequencies in the western ghats, india. Conserv Biol. 2004 Dec;18(6):1553–1561.

26. Dandawate CN, Desai GB, Achar TR, Banerjee K. Field evaluation of formalin inactivated Kyasanur forest disease virus tissue culture vaccine in three districts of Karnataka state. Indian J Med Res. 1994 Apr;99:152–158.

27. Kasabi GS, Murhekar MV, Sandhya VK, Raghunandan R, Kiran SK, Channabasappa GH, et al. Coverage and effectiveness of Kyasanur forest disease (KFD) vaccine in Karnataka, South India, 2005-10. PLoS Negl Trop Dis. 2013 Jan 24;7(1):e2025.

28. Kiran SK, Pasi A, Kumar S, Kasabi GS, Gujjarappa P, Shrivastava A, et al. Kyasanur Forest disease outbreak and vaccination strategy,Shimoga District, India, 2013-2014. Emerging Infect Dis. 2015 Jan;21(1):146–149.

29. Purse BV, Darshan N, Kasabi GS, Gerard F, Samrat A, George C, et al. Predicting disease risk areas through co-production of spatial models: The example of Kyasanur Forest Disease in India's forest landscapes. PLoS Negl Trop Dis. 2020 Apr 7;14(4):e0008179.

30. Boshell J. Kyasanur Forest disease: ecologic considerations. Am J Trop Med Hyg. 1969 Jan;18(1):67–80.

31. Morse LJ, Russ SB, Needy CF, Buescher EL. Studies of viruses of the tick-borne encephalitis complex. II. Disease and immune responses in man following accidental infection with Kyasanur Forest disease virus. J Immunol. 1962 Feb;88:240–248.

32. Ripoche M, Gasmi S, Adam-Poupart A, Koffi JK, Lindsay LR, Ludwig A, et al. Passive tick surveillance provides an accurate early signal of emerging lyme disease risk and human cases in southern canada. J Med Entomol. 2018 Jun 28;55(4):1016–1026.

33. Jameson LJ, Medlock JM. Tick surveillance in Great Britain. Vector Borne Zoonotic Dis. 2011 Apr;11(4):403–412.
